# Genomic characterization and assessment of pathogenic potential of *Legionella* spp. isolates from environmental monitoring

**DOI:** 10.3389/fmicb.2022.1091964

**Published:** 2023-01-12

**Authors:** Ema Svetlicic, Daniel Jaén-Luchoro, Roberta Sauerborn Klobucar, Carsten Jers, Snjezana Kazazic, Damjan Franjevic, Goran Klobucar, Brian G. Shelton, Ivan Mijakovic

**Affiliations:** ^1^Novo Nordisk Foundation Center for Biosustainability, Kongens Lyngby, Denmark; ^2^Department of Infectious Diseases (Sahlgrenska Academy) at the University of Gothenburg, Gothenburg, Sweden; ^3^PathCon Laboratories EU, Zagreb, Croatia; ^4^Laboratory for Mass Spectrometry and Functional Proteomics, Ruder Boskovic Institute, Zagreb, Croatia; ^5^Division of Zoology, Department of Biology, Faculty of Science, University of Zagreb, Zagreb, Croatia; ^6^Systems and Synthetic Biology Division, Department of Biology and Biological Engineering, Chalmers University of Technology, Gothenburg, Sweden

**Keywords:** *Legionella*, whole-genome sequencing, environmental monitoring, virulence factors, novel species

## Abstract

Several species in the genus *Legionella* are known to cause an acute pneumonia when the aerosols containing the bacteria from man-made water systems are inhaled. The disease is usually caused by *Legionella pneumophila*, but other species have been implicated in the infection. The disease is frequently manifested as an outbreak, which means several people are affected when exposed to the common source of Legionella contamination. Therefor environmental surveillance which includes isolation and identification of *Legionella* is performed routinely. However, usually no molecular or genome-based methods are employed in further characterization of the isolates during routine environmental monitoring. During several years of such monitoring, isolates from different geographical locations were collected and 39 of them were sequenced by hybrid *de novo* approach utilizing short and long sequencing reads. In addition, the isolates were typed by standard culture and MALDI-TOF method. The sequencing reads were assembled and annotated to produce high-quality genomes. By employing discriminatory genome typing, four potential new species in the *Legionella* genus were identified, which are yet to be biochemically and morphologically characterized. Moreover, functional annotations concerning virulence and antimicrobial resistance were performed on the sequenced genomes. The study contributes to the knowledge on little-known non-pneumophila species present in man-made water systems and establishes support for future genetic relatedness studies as well as understanding of their pathogenic potential.

## 1. Introduction

*Legionella* is a genus of Gram-negative bacteria naturally occurring in freshwater habitats where they thrive in mixed community biofilms and replicate within protozoan hosts (Mondino et al., 2020). Moreover, the bacteria from the *Legionella* genus have an ability to inhabit man-made water systems, such as air conditioning units (Al-Matawah et al., 2015), cooling towers (Lepine et al., 1998; Wüthrich et al., 2019), hot tubs (Yakunin et al., 2020), and drinking water systems (Springston and Yocavitch, 2017). Human infection can subsequently occur as a result of aspiration of aerosols containing *Legionella*. The infection can cause an acute bacterial pneumonia termed Legionnaires’ disease or a milder form, a flu-like non-pneumonic Pontiac fever (Mercante and Winchell, 2015). Water distribution systems therefore pose a risk for large outbreaks of *Legionella*-related disease. Thus, specific guidelines for buildings, especially healthcare facilities are imposed to minimize the potential for transmission of *Legionella*. Various parameters affect the presence of *Legionella* species, such as water hardness, temperature, corrosion scale, flow regimes, and biofilms (Pécastaings et al., 2010). Hence, guidelines are focused on proactive efforts to recognize and evaluate *Legionella* hazards and to prevent disease through source identification, risk assessment, and control, rather than acting after disease occurrence. The guidelines, apart from temperature regulations and prevention of water stagnation recommend routine testing for the presence of *Legionella* species (Berkelman and Pruden, 2017). Commonly used methods include traditional plate culture on Buffered charcoal yeast extract (BYCE) agar where presence of species from the *Legionella* genus is reported in colony forming units (CFU) per volume (Toplitsch et al., 2021). Although the *Legionella* genus comprises more than 70 species according to the List of Prokaryotic names with Standing in Nomenclature (LPSN; Parte, 2014), *Legionella pneumophila* is responsible for around 90% of clinical manifestations of Legionnaires’ disease. Next, the most commonly isolated *Legionella* species are *Legionella longbeachae* and *Legionella bozemanii* (Yu et al., 2002; Phin et al., 2014). Less frequent *Legionella* species isolated from humans are *Legionella micdadei* (Waldron et al., 2015), *Legionella feeleii* (Siegel et al., 2010), *Legionella dumoffii* (Joly et al., 1986), *Legionella wadsworthii* (Edelstein et al., 1982), and *Legionella anisa* (Vaccaro et al., 2016), which are associated with disease in immunocompromised patients. The non-pneumophila species in these samples are generally not well studied and therefore, despite their frequent isolation from water systems, not much is known about their pathogenic potential (Muder and Victor, 2002; Chambers et al., 2021). A major factor for limited availability of epidemiological data of non-pneumophila species is under-diagnosis due to mild symptoms or tests specifically targeting *L. pneumophila* such as frequently used urinary antigen test (Chambers et al., 2021). For the above-mentioned reasons, it was noted that correct identification and typing of strains isolated from patients and the environment is crucial for understanding the pathogenic role of bacteria belonging to the *Legionella* genus (Mazzotta et al., 2021; Du et al., 2022; Wroblewski et al., 2022). Whole-genome sequencing (WGS) analysis is a highly discriminatory method capable of assessing genetic relatedness among isolates and therefore constitutes an important tool for outbreak investigations as well as pathogen surveillance (Quainoo et al., 2017; Xu et al., 2021). WGS has been successfully applied in elucidating outbreak versus non-outbreak *L. pneumophila* strains (Reuter et al., 2013) and linking outbreaks to possible environmental sources (David et al., 2017). Furthermore, WGS-based characterization was used to explore the repertoire of effectors in 38 *Legionella* species providing important information for understanding the pathogenicity of the genus. Importantly, it was discovered that all sequenced *Legionella* species encode a conserved type IVB secretion system which is crucial for intracellular replication in the host. Conversely, large variation in secreted effectors was observed (Burstein et al., 2016).

Due to increased use of WGS for outbreak investigation, the number of genome sequenced *L. pneumophila* isolates is increasing. By comparison, genome sequences of non-pneumophila species present in the isolates are underrepresented. For example, National Center for Biotechnology Information (NCBI) currently hosts more than 3,800 *L. pneumophila* assemblies, while other *Legionella* species are represented with maximum 16 assemblies. This makes informative genome comparative studies especially difficult. Moreover, the *Legionella* genus is known for having a high genome diversity because of recombination and horizontal gene transfer (HGT) between different bacteria and even involving eukaryotes (Gomez-Valero and Buchrieser, 2019). HGT is an important evolutionary mechanism which enables bacteria to rapidly adapt to their environment which includes the acquisition of pathogenicity and/or antimicrobial resistance genes. It has been hypothesized that *Legionella* acquired virulence genes which enable human infection *via* HGT from protozoa, in which it replicates in the environment (Gomez-Valero and Buchrieser, 2013, 2019).

The aim of the study herein was to investigate the diversity, virulence potential, and antimicrobial resistance of *Legionella* sp. isolates obtained during environmental monitoring using WGS. Samples from different geographical locations were collected and confirmed to be *Legionella* by ISO 11731 procedures (ISO 11731:2017, n.d., Water quality—Enumeration of Legionella). Isolates were not connected to an outbreak, and 39 samples isolated from 15 countries were chosen randomly for WGS and further characterized in pangenome, virulence, and antimicrobial resistance analysis. Interestingly, in this relatively small sample size, four isolates were determined to be new species candidates based on genomic relatedness estimated by Average Nucleotide Identify (ANI) and *in silico* DNA–DNA hybridization. Two of the named four isolates showed genetic relatedness to *Legionella cherrii*, while the other two showed closest relation to *L. pneumophila*.

## 2. Materials and methods

### 2.1. Culture analysis and MALDI-TOF species identification

For this study, 39 isolates were randomly selected (fluorescent and non-fluorescent) from a collection of strains isolated over a period of 5 years (2016–2021) in different countries during environmental surveillance in building water systems. All isolates were characterized by culture methods described by ISO 11731:2017 and with application of procedures for the recovery of Legionella from the environment. All presumptive Legionella colonies were additionally confirmed on culture media; Buffered charcoal yeast extract (BCYE) agar and BCYE-cysteine agar according to ISO 11731-2017 in an accredited microbiological laboratory (EN ISO/IEC 17025: 2017; HAA 1550). The isolates were also subjected to matrix-assisted laser desorption/ionization time of flight mass spectrometry (MALDI-TOF MS) analysis with full extraction as previously described (Pečur Kazazić et al., 2019). Briefly, a loopful of a bacterial colony was suspended in 300 μl deionized water, vortexed, added 900 μl of absolute ethanol (Kemika, Croatia) to the suspension, and centrifuged at 13,000 rpm for 2 min. The supernatant was decanted and the pellet was resuspended by pipetting in the equal volume of 70% formic acid (Sigma Aldrich, Germany) and 100% acetonitrile (Fisher Chemical, Spain) and centrifuged at 13,000 rpm for 2 min. The supernatant was spotted onto a 96-spot polished steel target plate (Bruker Daltonik, Germany), dried at room temperature, and overlaid with 1 μl of 10 mg/ml alpha-4-cyano-4-hydroxycinnamic acid (CHCA, Bruker Daltonik, Germany) in 50% acetonitrile and 2.5% trifluoroacetic acid and allowed to dry.

Spectra were acquired in the positive linear ion mode in the mass range of 2–20 kDa. MBT Compass HT version 5.0 software (Bruker Daltonik) was used for spectra matching to a reference database version 11. Identification criteria were as follows: a log score of 2.00–3.00 indicated high-confidence species identification, a log score of 1.70–1.99 indicated low-confidence species identification, while a score of 0–1.69 was considered unreliable identification.

### 2.2. DNA extraction and sequencing

A single colony was picked from BYCE agar, resuspended in PBS and the pellet was washed three times. DNA was extracted from each sample using the DNeasy blood and tissue kit (Qiagen, Germany), RNase treatments and following the manufacturer’s instructions. Genomic libraries were prepared using Illumina Nextera DNA library prep according to the manufacturer’s protocol and were sequenced using the Illumina MiSeq system thereby obtaining paired-end 150 bp reads. Long reads were obtained by MinION Nanopore (Oxford Nanopore Technologies, United Kingdom). The DNA libraries for long-read sequencing were prepared using rapid 96 barcoding kit following the manufacturer’s instruction and sequenced using R9.4.1 flow cell. The run was performed until sufficient data yield was obtained. Basecalling was performed in real-time using Guppy 5.0.11 (nanopore) with high accuracy basecalling model and reads were exported as FastQ files for further analysis.

### 2.3. *De novo* hybrid assembly and annotation

Quality control and trimming of low-quality Illumina reads were conducted using fastp (v0.20.1) with default settings (Chen et al., 2018). Adapter trimming of long reads was performed using Porechop (v0.2.4)^1^ and the read quality was checked with Nanoplot v1.28.2 (De Coster et al., 2018). Illumina and Nanopore reads were assembled by hybrid *de novo* assembly using Unicycler (v0.4.1; Wick et al., 2017) with default settings. The quality of the assembly was checked with quast (v.5.2.0; Gurevich et al., 2013). The quality control reports were summarized using MultiQC (Ewels et al., 2016). Functional annotation of the *de novo* assembled genomes was done by Prokka (Galaxy v1.14.6; Seemann, 2014; Afgan et al., 2018). Contamination was assessed using checkM (v1.0.18, kBASE; Parks et al., 2015; Arkin et al., 2018) which checks quality of genome sequences of isolates, single cells, or genome bins from metagenome assemblies through comparison to an existing database of genomes included in the chekM tool.

### 2.4. Genome-based species identification

The initial typing of the assemblies was conducted by PubMLST (Jolley et al., 2012) to identify the closest related species from the database. The assemblies of type strains belonging to *Legionella* genus were downloaded from NCBI and crosschecked with LPSN database. The reference database was constructed from 59 type strain assemblies which are listed in Supplementary Table S1.

The 39 isolates sequenced in this study were matched to type strains assemblies using an alignment-free approach through the fastANI tool (Hayoun et al., 2020) to determine average nucleotide identity (ANI). Next, ANI based on BLASTn pairwise comparisons against *Legionella* genus type strains and isolates from this study were calculated with JSpeciesWS server (Richter et al., 2016). Calculation of genome-to-genome distance by *in silico* DNA–DNA hybridization (GGDC; Meier-Kolthoff et al., 2022) was also conducted for all 39 isolates against *Legionella* type strains.

The core-genome determination was derived from the 59 *Legionella* type strains that were available in NCBI (Supplementary Table S1), as well as the 39 genome assemblies produced in this study. For normalization purposes, all genomes were annotated with Prokka (Galaxy v1.14.6). The core genome was determined using several available tools and an in-house script as previously described (Jaén-Luchoro et al., 2020). In brief, the protein sequences of the genomes were compared (all vs. all) using the Basic Local Alignment Search Tool for Proteins (BLASTP; Altschul et al., 1990). Based on these results, groups of homologous proteins were formed, using the Get Homologues software. The threshold for homology was set to 70% similarity for at least 70% of the respective sequence. Alignments were concatenated, and then processed by GBLOCKS (Castresana, 2000). This final alignment was used to build a core-genome phylogeny, using the Maximum Likelihood algorithm with PhyML (Guindon et al., 2010) and the Approximate Likelihood-Ratio Test (aLRT; Anisimova and Gascuel, 2006).

Additionally, a complete 16S rRNA gene sequence phylogenetic tree was built using MEGA v7.0.26 (Kumar et al., 2016). Sequences were aligned using ClustalW and distance matrix was generated. The evolutionary distances were calculated using the Kimura-two parameter model (Kimura, 1980), and clustering analysis was performed by Neighbor-Joining (Saitou and Nei, 1987) Bootstrap parameter was set for 1,000 replications.

### 2.5. Virulence factors and antimicrobial resistance screening

The files containing proteins sequences of each isolate were screened for virulence and antimicrobial resistance (AR) genes using (Galaxy v.1.0.1).^2^ Virulence factors and AR genes were identified using ABricate (Seemann, 2022a). For this purpose, the Virulence factor database (VFDB; Chen et al., 2016) and Comprehensive Antibiotic Resistance Database (CARD; Jia et al., 2017) were used, respectively. In order to investigate the point mutations previously described in Legionella genus, target protein sequences encoded by genes gryA, gryB, parC, rpoB, rplD, and rplV were aligned to corresponding *Escherichia coli* K12 genes to have a consistent numbering amino acid positions. The alignments were visualized in Jalview (Troshin et al., 2011).

Sequence-based typing (SBT) of *L. pneumophila* is a discriminatory and reproducible method for genotyping clinical and environmental *Legionella* isolates. The method uses seven loci including five virulence genes (*flaA, pilE, mip, mompS*, and *proA*) and two housekeeping genes (*asd* and *neuA*). In this study, *in silico L. pneumophila* SBT was achieved using a freely available tool legsta v0.5.1 (Seemann, 2022b) which contains sequences and SBT profiles obtained from the Public Health England database.^3^

### 2.6. Detection of putative plasmids

Since many isolates from this study contained 2 or 3 contigs which were determined to be circular plasmid presence was suspected. The presence of plasmid-derived sequences in the assemblies was first checked using BLASTn against NCBI non redundant (nr) database.

### 2.7. Intra-species comparisons

Pangenome analysis was performed using the Bacterial Pan Genome Analysis Pipeline (BPGA tool; Chaudhari et al., 2016). The default clustering tool USEARCH (Edgar, 2010) was used to define orthologous clusters of protein sequences based on the default 50% sequence identity cut-off. The pan-genome functional analysis module was used to find the Clusters of Orthologous Groups of proteins (COGs) and map pathways using Kyoto Encyclopedia of Genes and Genomes (KEGG) database (Kanehisa and Goto, 2000). For *L. pneumophila*, additional typing was performed based on core single nucleotide polymorphism (SNP) phylogenetic tree which included isolates from thus study closely matching to *L. pneumophila* and complete genome sequences downloaded from NCBI. The core genome SNPs were derived by using kSNP4 program, based on k-mer analysis of genome sequences of each isolate (Gardner et al., 2015). The tree was inferred using the Neighbor-Joining method from the matrix containing SNP loci common to all isolates.

## 3. Results

### 3.1. Culture identification and MALDI-TOF typing

**Figure 1:**
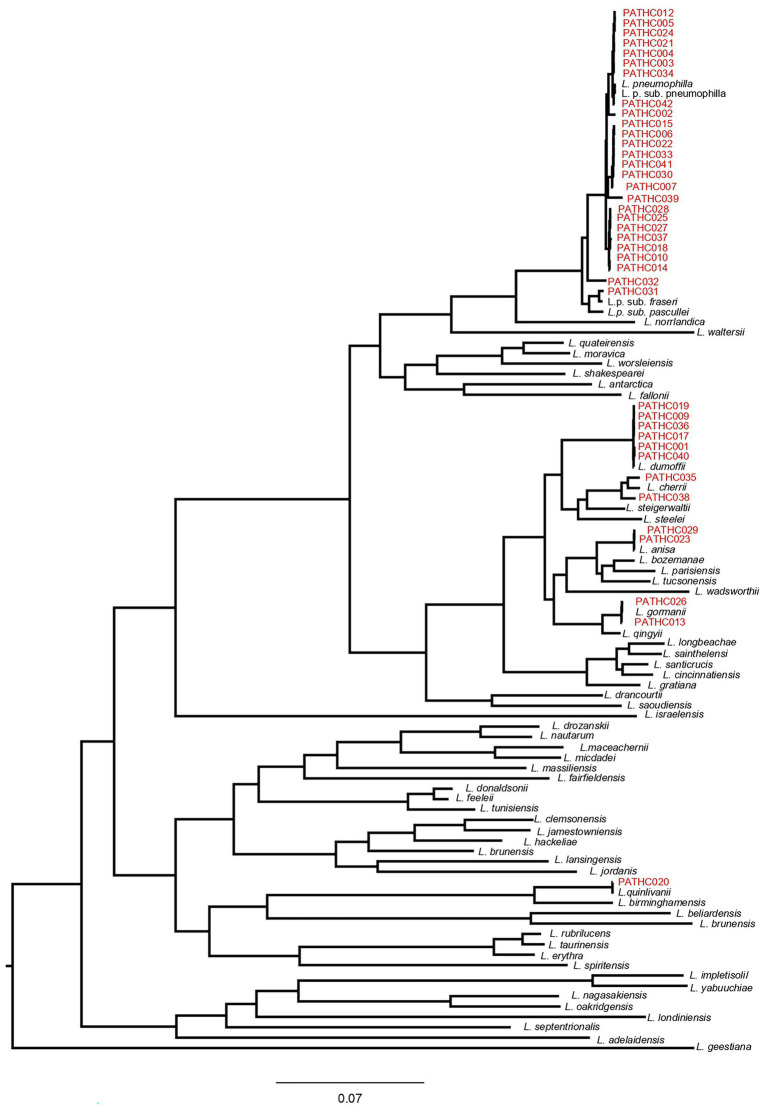
Core-genome dendrogram of *Legionella* type strains and the 39 newly sequenced *Legionella* isolates from this study. The core-genome was derived from 141 protein sequences encoded by single-copy genes. The tree was constructed, using Maximum Likelihood (ML) and the Approximate Likelihood-Ratio Test (aLRT).

During environmental monitoring of *Legionella*, numerous samples were collected in the period of 2016–2021. For the purpose of this study, 39 isolates representing 15 locations around the world were chosen for WGS randomly as shown in Table 1 After confirming that the isolates belonged to the *Legionella* genus by the culture method, the strains were typed by MALDI-TOF to identify the bacterial species (Table 1). Based on the MALDI-TOF analysis, 26 *L. pneumophila* strains and six strains of *L. dumoffii* were identified. Moreover, two strains were identified for each of the following species: *Legionella anisa*, *Legionella cherrii*, and *Legionella gormanii*. Moreover, one strain of *Legionella quinlivanii* was also identified (Table 1; Figure 1).

**Table 1:** Isolates selected for the study along with their country of origin, isolation source, results of MALDI-TOF typing, and PubMLST typing.

Sample ID	Isolation source	Country of origin	MALDI biotyper identification	MALDI score	PubMLST
PATHC001	Hot water tank	Saudi Arabia	*Legionella dumoffii*	2.25	*Legionella dumoffii*
PATHC002	Incoming water supply	Saudi Arabia	*Legionella pneumophila*	2.21	*Legionella pneumophila*
PATHC003	Hot water tank	Uganda	*Legionella pneumophila*	2.15	*Legionella pneumophila*
PATHC004	Calorifier tank	United Arab Emirates	*Legionella pneumophila*	2.43	*Legionella pneumophila*
PATHC005	Shower	Oman	*Legionella pneumophila*	2.25	*Legionella pneumophila*
PATHC006	Kitchen sink	Oman	*Legionella pneumophila*	2.04	*Legionella pneumophila*
PATHC007	Hot water tank	Albania	*Legionella pneumophila*	2.29	*Legionella pneumophila*
PATHC009	Shower	United Arab Emirates	*Legionella dumoffii*	2.37	*Legionella dumoffii*
PATHC010	Sink	United Arab Emirates	*Legionella pneumophila*	2.6	*Legionella pneumophila*
PATHC012	Jacuzzi	Ethiopia	*Legionella pneumophila*	2.33	*Legionella pneumophila*
PATHC013	Irrigation tank	United Arab Emirates	*Legionella gormanii*	2.35	*Legionella gormanii*
PATHC014	Irrigation tank	United Arab Emirates	*Legionella pneumophila*	2.3	*Legionella pneumophila*
PATHC015	Shower	Gabon	*Legionella pneumophila*	2.26	*Legionella pneumophila*
PATHC017	Shower	Oman	*Legionella dumoffii*	2.41	*Legionella dumoffii*
PATHC018	Incoming water supply	Djibouti	*Legionella pneumophila*	2.32	*Legionella pneumophila*
PATHC019	Pool shower	United Arab Emirates	*Legionella dumoffii*	2.29	*Legionella dumoffii*
PATHC020	Water tank	United Arab Emirates	*Legionella quinlivanii*	2.55	*Legionella quinlivanii*
PATHC021	Raw water tank	United Arabian Emirates	*Legionella pneumophila*	2.35	*Legionella pneumophila*
PATHC022	Decorative fountain	Croatia	*Legionella pneumophila*	2.34	*Legionella pneumophila*
PATHC023	Decorative fountain	Croatia	*Legionella anisa*	2.22	*Legionella anisa*
PATHC024	Jacuzzi	Ethiopia	*Legionella pneumophila*	2.37	*Legionella pneumophila*
PATHC025	Shower	Guinea	*Legionella pneumophila*	2.3	*Legionella pneumophila*
PATHC026	Cold water storage tank	Guinea	*Legionella gormanii*	2.3	*Legionella gormanii*
PATHC027	Sink	Guinea	*Legionella pneumophila*	2.27	*Legionella pneumophila*
PATHC028	Sink	Guinea	*Legionella pneumophila*	2.24	*Legionella pneumophila*
PATHC029	Shower	Turkey	*Legionella anisa*	2.22	*Legionella anisa*
PATHC030	Bathtub	Austria	*Legionella pneumophila*	2	*Legionella pneumophila*
PATHC031	Hot water tap	United Arab Emirates	*Legionella pneumophila*	2.37	*Legionella pneumophila*
PATHC032	Hot water tap	United Arab Emirates	*Legionella pneumophila*	2.19	*Legionella pneumophila*
PATHC033	Cooling tower	United Arab Emirates	*Legionella pneumophila*	2.32	*Legionella pneumophila*
PATHC034	Pool	Nigeria	*Legionella pneumophila*	2.29	*Legionella pneumophila*
PATHC035	Sink	Nigeria	*Legionella cherrii*	2.3	*Legionella cherrii*
PATHC036	Jacuzzi	United Arab Emirates	*Legionella dumoffii*	2.33	*Legionella dumoffii*
PATHC037	Jacuzzi	United Arab Emirates	*Legionella pneumophila*	2.36	*Legionella pneumophila*
PATHC038	Hose bib	Cruise Ship	*Legionella cherrii*	1.84	*Legionella cherrii*
PATHC039	Shower	Cruise Ship	*Legionella pneumophila*	2.22	*Legionella pneumophila*
PATHC040	Shower	Ethiopia	*Legionella dumoffii*	2.23	*Legionella dumoffii*
PATHC041	Incoming water supply	Ghana	*Legionella pneumophila*	2.2	*Legionella pneumophila*
PATHC042	Shower	Ghana	*Legionella pneumophila*	2.25	*Legionella pneumophila*

### 3.2. General characteristics of the hybrid *de novo* assembly

Overall, 27 out of 39 genomes were determined to be closed/circular with several assemblies having more than one circular contigs indicting a presence of plasmids. The total size of the assemblies ranged from 3.3 to 4.7 Mb with average being 3.7 Mb. The average N50 value was 3.4 Mbp, showing a satisfied completeness quality of the assemblies. The functional annotation results provided by Prokka showed there are on average 3,253.6 coding sequences, 9.1 rRNAs and 43 tRNAs. The general overview of assemblies for each isolate can be found in Supplementary Table S2. New isolate assemblies were not substantially contaminated, with contamination levels ranging between 0 and 1.72% as determined by CheckM Moreover, with the same tool, genome completeness was assessed, and all isolates showed 100% completeness except isolate PATHC035, which was 99.71% complete. The shorter circular contigs were assessed by Unicycler metrics and the contigs which were determined circular were blasted against NCBInr database. The sequences which matched to the plasmid sequences present in the database with minimum 80% identity were assigned as putative plasmids. Overall, at least one putative plasmid was identified in 29 isolates, while eight isolates had two putative plasmids (Table 2).

**Table 2:** Average general characteristics of the hybrid assembly of isolates sequenced in this study.

	Value range for all isolates	Average value for all isolates
Total length bp	3,332,865–4,486,832	3,669,645.4
No of contigs	1–55	4.3
GC content %	38.03–42.98	38.7
N50 bp	235,662–4,267,150	3,373,580.9
Number of coding sequences	2,934–3,996	3,253.6
No of rRNAs	6–12	9.1
No of tRNAs	42–44	43.0

### 3.3. Strain typing based on whole-genome sequencing

The initial strain typing was done by using public databases for molecular typing and microbial genome diversity, also known as PubMLST (Jolley et al., 2012). The tool uses Ribosomal Multilocus Sequence Typing (rMLST) approach, which accepts genome assemblies and catalogues a variation of bacterial ribosome protein subunit (rps gene) for the purpose of bacterial species identification. PubMLST typing provided closest match identification to all 39 isolates, matched with the species identifications performed by MALDI-TOF as shown in Table 1, and the majority of isolates (26) were identified as *L. pneumophila*, six isolates were identified as *L. dumoffii*, two isolates as *L. anisa*, *L. cherrii*, and *L. gormanii,* and one isolate as *L. quinlivanii*. The results of the PubMLST typing are shown in Table 1.

Next, various genomic relatedness tests were performed on the assemblies as detailed below and summarized in Table 3. ANI of isolates was firstly determined by an alignment-free approach using fastANI matching against assemblies of the *Legionella* type strains. This approach provided rapid comparison between isolates and type strains which confirmed the results of MALDI-TOF and PubMLST when it comes to the most related species in the *Legionella* genus. However, three isolates PATHC0032, PATHC0035, and PATHC0038 exhibited ANI lower than 95% with the closest related *Legionella* species (Table 3). Next, to further investigate if these isolates could represent a different taxa from the currently known bacterial species in the *Legionella* genus, ANIb and digital DNA–DNA hybridization (dDDH) were performed using JspeciesWS and GGDC, respectively, against all *Legionella* type strain genome sequences. Additionally, a complete 16S rRNA gene-based phylogenetic tree was constructed.

**Table 3:** Summary of *in silico* novel species proof test for the isolates determined to be new species candidates.

Isolate name	Closest hit	fastANI	ANIb	dDDH
*Legionella* sp. *PATHC032*	*Legionella pneumophila*	93.51	93.09	52.3
*Legionella* sp. *PATHC039*	*Legionella pneumophila*	95.97	95.8	68.1
*Legionella* sp. *PATHC035*	*Legionella cherrii*	94.30	93.97	56.2
*Legionella* sp. *PATHC038*	*Legionella cherrii*	93.77	93.94	54.7

Closest hit columns indicate the species most closely related as determined by fastANI, ANIb and Digital-DNA–DNA Hybridization (dDDH). For ANI-based search, a novel species threshold is similarity below 95%, while dDDH has a threshold of 70%.

The16S rRNA phylogenetic tree was constructed based on 1,423 nucleotide positions of the gene and is shown in Supplementary Figure S1. The similarity matrix extracted from the alignment used for constructing the tree is in Supplementary Information Sheet S2. This phylogenetic tree showed *Legionella* sp. PATHCC032 and PATHC039 clustering with the *Legionella pneumophila* subspecies, with which both shared more than 99% of sequence similarity*. Legionella* sp. PATHC035 clustered together with *L. cherrii* (99.6% of sequence identity) and shared more than 98.5% of sequence similarity with four *Legionella* species. For its side, *Legionella* sp. PATHC038 shared more than 98.7 of sequence similarity with six *Legionella* species, being *Legionella anisa* the most similar sequence (99.1%).

**Figure 2:**
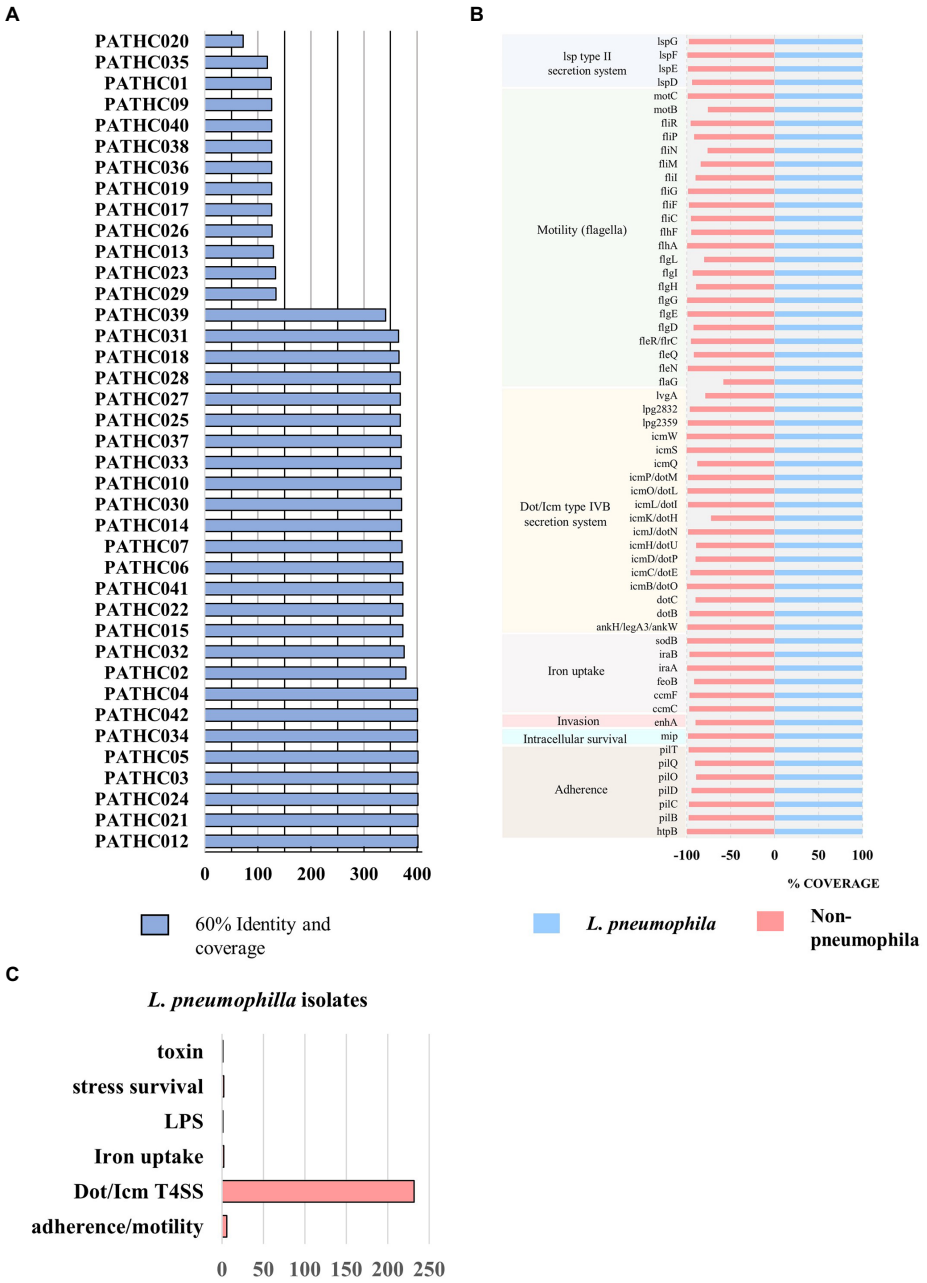
Results of the virulence factor search against VFDB. Coverage represents percentage of the gene covered and identity percentage of exact nucleotide matches. **(A)** The number of putative virulence factors in the isolates displaying 60% coverage and 60% identity to virulence factors in the VFDB. **(B)** Virulence genes shared among all isolates sequenced in this study. **(C)** Biological role of virulence factors only found in *Legionella pneumophila* isolates compared to non-pneumophila isolates.

The ANI values obtained with JSpeciesWS are in accordance with alignment-free fastANI results, and isolates PATHC032, PATHC035, and PATHC038 showed ANIb results below the species threshold of 95% with respect to the most closely related species for each isolate (Table 3). The dDDHs of all isolates were performed with BLAST+ as local alignment tool, as recommended. The results of the recommended formula 2 were below 70% for the isolates previously distinguished as novel (PATHC032, PATHC035, and PATHC038) and for an additional isolate PATHC039 which has borderline fastANI and ANIblast values of 95.97 and 95.8, respectively. These genomic comparison results support the hypothesis that, at least, isolates PATCH32, PATCH 35, and PATCH38 may represent three new species. However, more in-depth analysis should be done to demonstrate and properly describe the putative new taxa. Core genome analysis was conducted based on a comparative analysis of 59 *Legionella* type strains with available genome sequences and the 39 newly assembled genomes of strains from this study. BLAST analysis of all proteins against all was performed, and homologous proteins were clustered (70% of similarity in, at least, 70% of the sequence). From all homologous clusters, 267 core proteins were present in all genomes, of which 141 proteins were single-copy core proteins according to the consensus of three homologous clustering algorithms (BDBH, COGT, and OrthoMCL). These 141 proteins were concatenated and aligned. From the resulting alignment, a total of 40,252 amino acid homologous positions were used to build the core-genome tree, using Maximum Likelihood (ML) and the Approximate Likelihood-Ratio Test (aLRT; Figure 2). All strains closely related to *L. pneumophila* species as determined by the ANI and GGDC approach clustered with *L. pneumophila* and respective subspecies. However, it can be seen that isolates PATHC032 and PATHC039 form longer branches in the *L. pneumophila* cluster. Furthermore, isolates PATHC035 and PATHC038 are clustering with *L*. *cherrii* species, but exhibit longer branches compared to the same species clusters. The branch lengths are supported by ANI and GGDC values for those isolates.

### 3.4. Virulence potential of isolates and antimicrobial resistance

**Figure 3:**
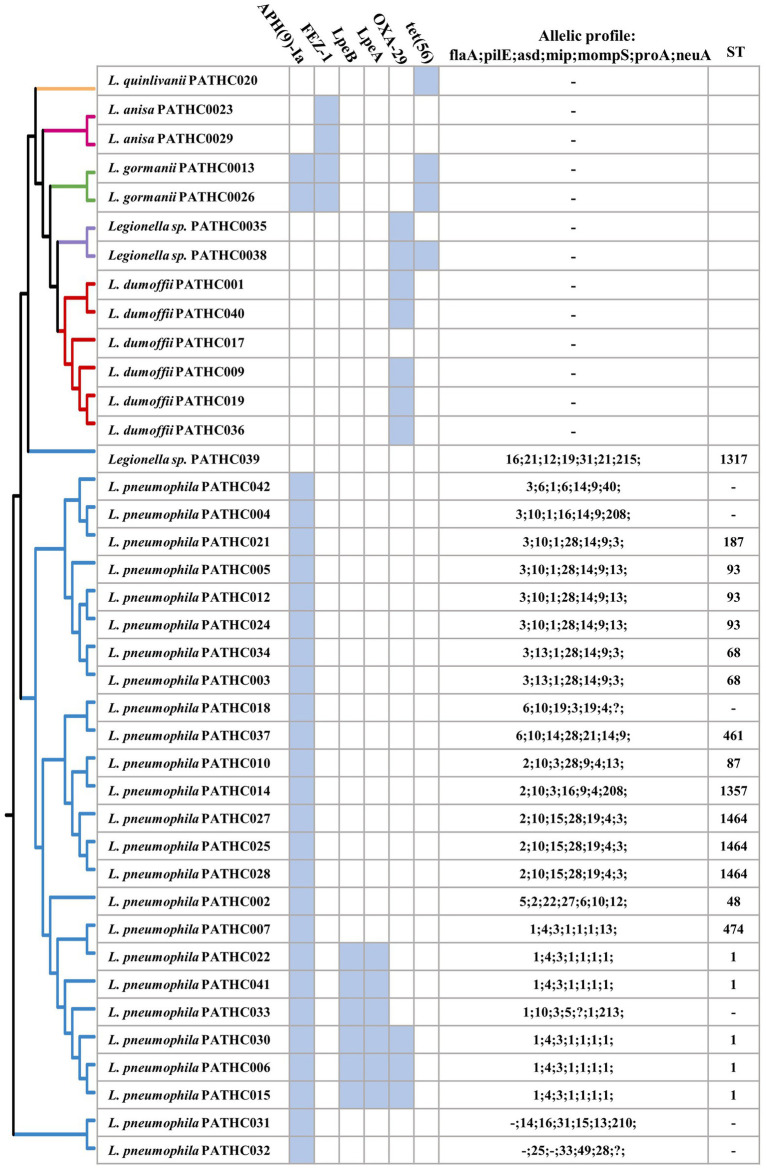
Antimicrobial resistance genes and sequence-based typing of *Legionella* isolates in this study grouped by pangenome tree constructed using the BPGA pipeline. Antimicrobial genes were predicted *in silico* searching the CARD database. Sequence-based typing was conducted *in silico* utilizing legsta tool which contains sequence base type profiles from the Public Health England database.

Virulence factors have been identified in the assemblies by searching the curated and comprehensive database of bacterial virulence factors (VFDB) with 60% coverage and 60% identity as thresholds. The thresholds were set to 60% due to VFDB containing only virulence factor sequences of *L. pneumophila* and relying on partly orthologous genes when it comes to searching other *Legionella* species. The results of the search showed the presence of 300–400 putative virulence genes in the isolates identified as *L. pneumophila* and up to 134 putative virulence factors identified in non-pneumophila species as shown in Figure 2A. Even though non-pneumophila species are not found in the VFDB, many virulence factors were discovered to be orthologous to the *L. pneumophila* strains. In total, 59 putative virulence genes were shared among all isolates, although in some case, the gene coverage was decreased in non-pneumophila species (Figure 2B). The shared virulence genes predominantly belong to the Dot/Icm Type IV secretion system and to flagellar structures. Moreover, a virulence factor known as the macrophage infectivity potentiator (*mip*) which is responsible for intracellular survival was found in both *L. pneumophila* and non-pneumophila species with high coverage of 100 and 98.6%, respectively. The gene *htpB* was found in all isolates with a minimum 99.8 coverage. This gene encodes for heat shock protein Hsp60 which is important for cell attachment and invasion. Other common genes were involved in adherence, iron uptake, and a secretion system called Lsp type II secretion. On the other hand, 250 predicted virulence factors were identified exclusively in isolates closely matching to *L. pneumophila*. The virulence factors were grouped into categories based on their biological function and it was determined that majority belong to T4SS secreted effectors (Figure 2C). T4SS system in *Legionella* pathogenesis involves several gene clusters. The locus containing genes *IimW* and *icmX* was found to be involved in establishment of *Legionella*-containing vacuole inside macrophages. Both genes were found in the *L. pneumophila* isolates; however, *icmX* was missing from the non-pneumophila isolates. The same situation can also be seen for other dot/icm genes, such as *icmL/dotI* and *sidJ* that encoded for T4SS effectors, as well as gene *rtxA*, which produces a membrane acting toxin. Only nine genes were found in non-pneumophila isolates that were not detected in *L. pneumophila* isolates. A closer inspection of the genes showed that they were orthologous to genes present in other bacterial genera (as it is shown in Table 4). A More in-depth complete information analysis of virulence genes found per isolate is available in Supplementary Information Sheet S1. AR genes were also searched in the assemblies against a CARD database. In total, six antimicrobial resistance genes were identified in the isolates as seen in Figure 3. There were three genes predicted in *L. gormanii* strains (PATHC013 and PATHC026) which included chromosomal-encoded aminoglycoside phosphotransferase (APH(9)-Ia), Ambler class B metallo-β-lactamase (FEZ-1) and tetracycline-resistant ribosomal protection protein (tet(56)). Most of the 25 strains belonging to *L. pneumophila* had only the aminoglycoside O-phosphotransferase *aph(9)-Ia* resistance gene, however six isolates had 2–3 additional resistance genes as it can be seen on Figure 3. These included the APH(9)-Ia, macrolide resistance determinants LpeAB as well as β-lactamase OXA-29. Interestingly, five out of the six resistance gene isolates belonged to ST1. It is worth noting that in the isolate PATHCO039, a potential novel species, no known AR genes were found, even though it is closely related to *L. pneumophila.* Isolates determined to be *L. dumoffii* contained the gene encoding producing a β-lactamase OXA-29, while metallo-β-lactamase FEZ-1 was found in *L. anisa* isolates. Additionally, point mutations known to cause antibiotic resistance in Legionella were searched. These include mutations in proteins encoded by genes gryA, gryB, parC, rpoB, rplD, and rplV (Nielsen et al., 2000; Jonas et al., 2003; Almahmoud et al., 2009; Descours et al., 2017). However, the named point mutations were not identified in the isolates as it is shown in Supplementary Table S4 and Supplementary Figures S9–S14.

**Table 4:** Virulence factors (VF) found in exclusively non-pneumophila isolates.

VF category	Gene name	Orthologous with gene from	Detected in isolates
capsule	*cap8E*	*Staphylococcus*	PATHC029, PATHC023, PATHC017, PATHC019, PATHC036, PATHC040, PATH00C9, PATHC001
LPS	*clpP*	*Listeria monocytogenes*	PATHC032, PATHC039, PATHC017, PATHC019, PATHC036, PATHC040, PATH00C9, PATHC001, PATHC026, PATHC013, PATHC038
Capsule	*fcl*	*Campylobacter*	PATHC020
LPS	*gmd*	*Yersinia enterocolitica*	PATHC029, PATHC023, PATHC020
LPS	*manB*	*Yersinia enterocolitica*	PATHC020
LPS	*rffG*	*Haemophilus influenzae*	PATHC029, PATHC023, PATHC020
Capsule	*tviB*	*Salmonella enterica*	PATHC028, PATHC035
T6SS	*vipB/mglB*	*Vibrio cholerae*	PATHC026, PATHC013, PATHC017, PATHC019, PATHC036, PATHC040, PATH00C9, PATHC001
LPS	*wzt*	*Brucella melitensis*	PATHC020

The bacterial genus/species to which the queried sequences matched is listed. LPS, Lipopolysaccharide; T6SS, Type 6 Secretion System.

Based on an SBT analysis of the *L. pneumophila* (and related) environmental isolates, 20 isolates were assigned to 11 distinct STs, whereas for six isolates the sequence type could not be assigned. The most prevalent type was sequence type 1 (ST1) with 19.2% isolates (5/26) followed by ST1464 and ST93 with 11.5% (3/26).

### 3.5. Intra-species genomic comparisons

**Figure 4:**
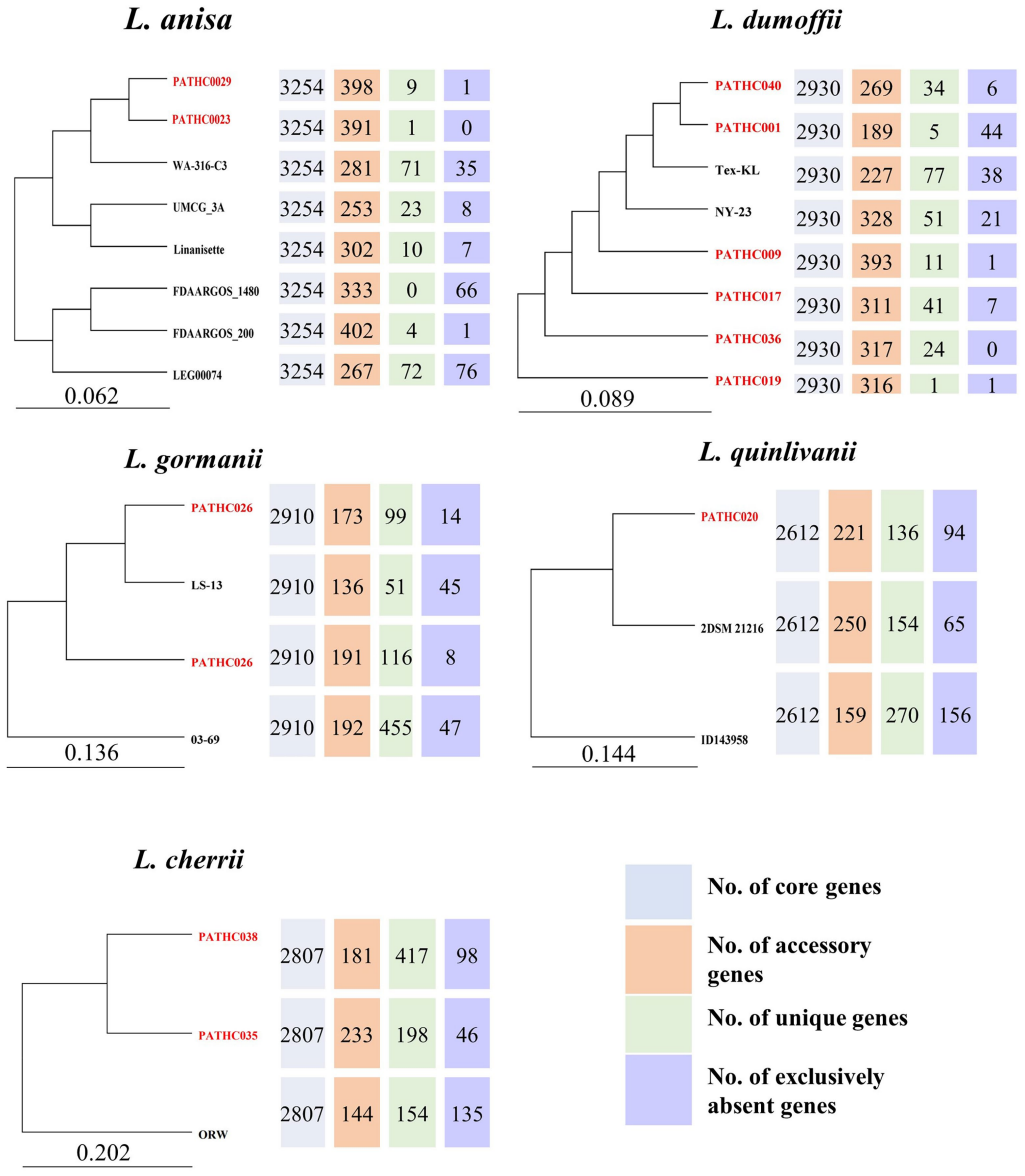
Comparative inter-species genome analysis of non-pneumophila genomes available from NCBI and genomes sequenced in this study. The pangenome trees were constructed with Bacterial Pan Genome Analysis Pipeline (BPGA). The number of core, accessory, unique, and exclusively absent genes was determined by the same pipeline.

A pangenome analysis was conducted for species found in this study based on our genome assemblies as well as those available in the GenBank. The BPGA software identified core, accessory, unique, and exclusively absent genes. The number and sequences of exclusively absent genes is a unique feature of the BPGA tool and it represents genes present in all but one genome from a study. Comparative functional analysis was performed based on COG and KEGG pathways depicted in the Supplementary material. Since the genome assemblies for non-pneumophila species generally are limited, a pangenome analysis was previously only conducted for *L. anisa* (Fleres et al., 2018). The core genome of *L. anisa* based on eight strains is 3,254 coding sequences which on average corresponds to 90.2% of the genome. According to the pangenome phylogenetic tree, the strains isolated in this study PATHC023 and PATHC029 showed the greatest similarity to each other and also contained a relatively small number of unique genes (1 and 9). According to KEGG, the majority of the unique genes were part of replication and repair mechanisms and signal transduction (Supplementary Figure S2). The core genome size of the 8 *L. dumoffii* strains was 2,930 genes (on average 90.1% of the genome), while the number of accessory genes ranged from 189 to 328. The largest number of unique genes was found in Tex-KL strain (77), while exclusively absent genes were found in PATHC001 in the highest number (44). Genes determined to be unique in one strain were highly represented in folding sorting and repair, signal transduction and replication and repair pathways. Accessory genes were more associated with energy metabolism, membrane transport and signal transduction (Supplementary Figure S3). On average 89.4% of the genes in the *L. gormanii* genome were part of the core genome where the core genome size was 2,907 genes. The isolate from this study PATHC026 was more closely related to the type strain LS-13 than the remaining two *L. gormanii* isolates. Unique genes were enriched in energy metabolism, while accessory genes were more associated with lipid metabolism and signal transduction pathways (Supplementary Figure S4). The core genome size of three *L. quinlivanii* strains was 2,612 genes (average of 86.8% of all genes). Accessory genes were distributed in amino acid and carbohydrate metabolism as well as membrane transport (Supplementary Figure S5). Since the two isolates PATCH035 and PATHC038 were determined to be closely related to *L. cherrii*, the pangenome analysis was conducted for the *L. cherrii* available genome. The core genome size of the three isolates was 2,781 genes (average of 86.5% of genes). Unique and accessory genes were, as for other non-pneumophila strains in this study, associated with energy metabolism and signal transduction (Supplementary Figure S6). The pangenome analysis was also conducted for *L. pneumophila* strains sequenced in this study together with closed genomes available from the NCBI. The pangenome analysis included 132 genomes and the results are shown in Supplementary Table S4 as well as on the pangenome phylogenetic tree in Supplementary Figure S7. The core genome size was determined to be 1,938 genes, or on average 65% of the genome. The isolate PATHC039 exhibited the highest number of unique genes (*n* = 104), followed by isolates PATHCO018 and Lansing3 (*n* = 54), and isolate PATHC032 (*n* = 48). The highest number of exclusively absent genes was also found in the isolates PATHC035 (*n* = 14) and PATHC032 (*n* = 16). Unique and accessory genes were mostly associated with energy replication, recombination and repair (Supplementary Figure S8). The phylogenetic tree based on the 25,011 core genome SNPs demonstrated that strains PATHC032 and PATHC039 are phylogenetically distant compared to the *L. pneumophila* strains included in the core SNP analysis as shown in Supplementary Figure 15. Majority of isolates have the number of SNPs ranging between 1 and 108, however, the named isolates PATHC032 and PATHC035 have 2,843 and 1,029, respectively (Figure 4).

## 4. Discussion

Although *L. pneumophila* is the most common and severe pathogen in the *Legionella* genus, numerous non-pneumophila species have been isolated from patients on at least one occasion. The progress of WGS has enabled insight into pathogen’s virulence mechanisms, antibiotic resistance as well as evolutionary mechanisms (Punina et al., 2015). The usefulness of WGS has been shown in *L. pneumophila* outbreak studies, which enabled typing and subtyping of the *Legionella* genus as well insight into evolutionary mechanisms (Qin et al., 2016; Kozak-Muiznieks et al., 2018). However, this has also led to an over-representation of complete genome sequences for the clinically more relevant *L. pneumophila*. Consequently, inter-species comparative genomics for non-pneumophila species are lacking due to low numbers of representative genome sequences. Thus, one aim of this study was to further characterize isolates identified as *Legionella* sp. during environmental monitoring. The samples confirmed to be *Legionella* sp. by culture method were subsequently sequenced to produce high-quality genome assemblies. Moreover, the aim of this study was to explore pathogenic potential of the isolates as well as diversity on the genome level. Utilizing hybrid *de novo* assembly, 28 out of 39 genomes were closed and the plasmid presence was indicated in 29 isolates. Although 11 genomes were not closed, they were estimated to be complete by checkM on the basis of housekeeping genes expected to be present in the isolates. The genome relatedness was elucidated by applying ANI-based tool (fastANI and ANIb) and dDDH which revealed that three isolates (32, 35, and 38) could potentially be new species based on a 95 and 70% similarity threshold for ANI and dDDH, respectively (Chun and Rainey, 2014). However, the isolate PATHC039 exhibited an ANI value higher than 95% but a dDDH value, which was previously appointed as conclusive when it comes to borderline values, of 68.1% (Chun and Rainey, 2014). Nevertheless, ANI values previously showed large genomic distances among *L. pneumophila* subspecies, markedly below 95% for subsp. *fraseri* and subsp. *pascullei* (Kozak-Muiznieks et al., 2018). High similarities in 16S rRNA gene are over the species threshold (98.7%; Stackebrandt and Ebers, 2006), and do not allow to identify the isolates as putative new species due to well-known limitations of this gene (Fox et al., 1992) according to which a high similarity of 16S rRNA sequence not always determines that two strains belong to the same species. The isolates were also identified with MALDI-TOF technique, which provided to be an accurate tool for species identification in this study. However, the named technique was previously not specific if the isolates analyzed were not contained in the database and resulted in several misidentifications (Pascale et al., 2020). When identifying novel species, this should be taken in account. Genomic analysis helps to overcome these limitations, allowing for a more comprehensive analysis. To confirm that the isolates belong to a novel species or a subspecies, a detailed characterization of morphological, physiological, and biochemical properties should be provided along with submission of strains to culture collections (Tindall et al., 2010) but this was beyond the scope of this study.

In addition to accurately typing bacterial isolates, WGS was proposed as a method for phenotype prediction especially when it comes to predicting virulence or antibiotic resistance (Balloux et al., 2018; Mason et al., 2018; Rokney et al., 2020). The common challenge of this approach is a need for a comprehensive and curated database which is representative of a large number of bacterial species. Regarding *L*. *pneumophila*, the VFDB contains virulence factors from five different strains, thus in this study, several hundred virulence genes were detected in the isolates closely related to *L. pneumophila* with a high gene coverage (>60%) and a high nucleotide similarity (>60%). A substantial amount of virulence factors were matched to non-pneumophila species (between 72 and 134). It is apparent that both pneumophila and non-pneumophila isolates from this study contain genes belonging to T4SS and T2SS. The T2SS is required for intracellular replication within macrophages and protozoa (Chauhan and Shames, 2021) It is hypothesized to be present in all Legionella species, which can be observed in the isolates from this study whereas all isolates harbored genes belonging to Lsp type II secretion system genes including *pilD* and *lspCDEFGHIJKLM*. For the T4SS, it was already observed that the Dot/Icm T4SS is highly conserved and present in all species regardless of diversity between genomes (Gomez-Valero and Buchrieser, 2013). However, T4SS are present in strains with varying degree which is also apparent in the genome of the strains sequenced in this study. The influence of these genes on pathogenicity seems to be host-dependent whereas mutation of certain genes impaired growth of Legionella in different amoebas (Park et al., 2020). In the named study, it was shown that by infecting macrophages *in vitro*, with silenced genes *mavN/iroT, sdhA, ravY,* or *lpg2505* impairs growth in macrophages. In the isolates from this study, *mavN* gene was found in all isolates; however, in non-pneumophila isolates, the gene identity was maximum 67%. In contrast, *sdhA, lpg2505, lpg1751,* and *ravY* were only found in *L. pneumophila* isolates including PATHCO032 and PATHC039, while the named gene was not identified in non-pneumophila isolates with the 60% identity threshold. In addition, mavQ and mavO were determined responsible for Legionella replication within macrophages in the previous study (Park et al., 2020), but they were not identified in any of the isolates from this study. While the assumptions on pathogenicity can be made based on the absence of the fewer number of the genes responsible for pathogenicity, conclusions can only be drawn from experimental data due to diverse nature of Legionella genus.

While the majority of those virulence factors were homologous *to L. pneumophila*, several were highly similar to other species including *Yersinia enterica* and *Vibrio cholerae.* This is not surprising, considering HGT from other bacterial species is recognized as a source of diversity in the *Legionella* genus (Gomez-Valero and Buchrieser, 2013).

Legionnaires’ disease is usually treated with macrolides and fluoroquinolones. Although AR in *Legionella* infections has not been a major concern, resistance to erythromycin, ciprofloxacin, rifampin, and azithromycin in *L. pneumophila* has been noted (Jia et al., 2019). Aminoglycoside O-phosphotransferase *aph(9)-la* gene responsible for spectinomycin resistance (Suter et al., 1997) was detected in all *L. pneumophila* and in *L. gormanii* isolates in this study. Isolate PATHC039, suspected to be a novel species did not contain this gene. This chromosomal-encoded gene was previously found in several *L. pneumophila* isolates, but it is not considered a risk due to the fact that Legionnaires’ disease is rarely treated with spectinomycin (Fong et al., 2010). The class D β-lactamase OXA-29 and a metallo-β-lactamases FEZ-1, found in several *L. dumoffii* isolates, are involved in bacterial resistance to β-lactams such as penicillin. A gene coding for FEZ-1 was previously described in *L. gormanii* as quite divergent from other similar β-lactamase (Boschi et al., 2000; Franceschini et al., 2001). Having both genes FEZ-1 and OXA-39 means the strains can efficiently hydrolyze different β-lactam substrates as it is observed in other pathogens which harbor multiple β-lactamase (Franceschini et al., 2001) The resistance proteins LpeA and LpeB form together an efflux pump conferring resistance to macrolide antibiotics. It was previously found in a subpopulation of *L. pneumophila* isolates exhibiting reduced susceptibility to azithromycin (Natås et al., 2019).

The point mutations previously associated with resistance to fluoroquinolones (Almahmoud et al., 2009), macrolides (Descours et al., 2017), and rifampicin (Nielsen et al., 2000) were not detected in any of the isolates sequenced in this study. The mutations were mostly associated with clinical isolates in the named study thus it could be that the environmental isolates from this study did not acquire mutations because of the lack of antibiotic selection pressure.

The subpopulation was associated with Sequence type 1 and Serogroup 1. Similarly, this can be observed in the isolates in this study where sequence type 1 isolates mostly contain lpeA and lpeB complex. Although, indicative, genome analysis should be confirmed with experimental data to confirm the antimicrobial resistance. Sequence-based typing is a genotyping approach developed by The European Working Group for *Legionella* Infection (EWGLI) involving PCR-amplification and sequencing of seven genes. Recently, the approach has been applied *in silico*, meaning that WGS data can be used for SBT and be associated with already known data. By comparing the sequence types with AR, it was confirmed that isolates harboring the *lpeA* and *lpeB* genes belong to ST1 and thus could be less susceptible to azithromycin (Massip et al., 2017).

Pangenome analyses focused on *L. pneumophila* have been previously performed on clinical and environmental isolates (Qin et al., 2016; Mercante et al., 2018; Ricci et al., 2022) The similar number of genes included in the core genome was obtained in this study, even though this study include by far the most *L. pneumophila* genomes which indicates the core genome has stabilized. The functional annotation shows that unique and accessory genes were required for replication, recombination, and repair, which is also in accordance with mentioned pangenome studies which suspect these genes were acquired by HGT. To compare non-pneumophila genomes, all available genomes were downloaded from NCBI. Since the downloaded genomes contained type strains sequenced from different laboratories, only unique strains/isolates were included in the pangenome analysis. This however decreased the sample size and made the pangenome analysis less informative. Nevertheless, the analysis contributed to the understanding of the number and function of genes shared among the species. The non-pneumophila strains exhibited a conserved genome, considering the core genome accounts for more than 85% of the total genome and the strains included in the pangenome study originated from different geographical locations. The conserved genome within species was also shown in pangenome of *L. pneumophila* (Gomez-Valero et al., 2011) and *L. anisa* (Fleres et al., 2018) strains from previous research. The named pangenome study of *L. pneumophila* also found a diverse accessory genome consisting of about 300 genes mostly found on mobile genetic elements. The number of accessory genes in strains *L. dumoffii* and *L. anisa* is similar to that of *L. pneumophila*; however, it is decreased in the remaining strains (*L. gormanii, L. quinlivanii*, and *L. cherrii*) whereas the unique gene number is higher. This could be explained by the low number of strains used for the analysis, it should be expected that with the addition of more genomes the number of unique genes should decrease.

## 5. Conclusion

The presented study characterized genomes from 39 *Legionella* isolates collected during environmental surveillance of man-made water systems. By employing genome-based typing of strains, four possibly new Legionella species were identified among the isolates. In addition, all strains were screened for virulence and antimicrobial resistance factors, offering insight into their pathogenic potential. High-quality genome assemblies were generated for the isolates, thus contributing to the limited genome information on non-pneumophila species. The data can be used for further research on pathogenicity of non-pneumophila species, but also for comparison between clinical and environmental isolates of *L. pneumophila*. Genome characterization offers knowledge on the risk of the potential *Legionella* outbreak caused by virulent strains, which could be crucial when implementing control measures.

## Data availability statement

The datasets presented in this study can be found in online repositories. The names of the repository/repositories and accession number(s) can be found at: https://www.ncbi.nlm.nih.gov/, PRJNA894337.

## Author contributions

ES performed sample preparation and data analysis, drafted the manuscript, and prepared figures and tables. DJ-L supervised the study, performed data analysis, prepared figures, and revised manuscript. RK designed the experiments, collected and isolated the strains, performed culture experiments, and drafted the manuscript. CJ supervised the study and wrote and revised the manuscript. SK performed MALDI-TOF analysis. DF and GK performed conceptualization, supervision, and revision of the manuscript. BS supervised the study and enabled sample collection. IM supervised the work helped revise the manuscript and acquired funding. All authors contributed to the article and approved the submitted version.

## Funding

The study has received funding from the European Union’s Horizon 2020 research and innovation program under the Marie Skłodowska-Curie grant agreement No 955626 (to IM) and the Novo Nordisk Foundation grant NNF20CC0035580 (to IM).

## Conflict of interest

RK and BS were employed by company PathCon Laboratories EU.

The remaining authors declare that the research was conducted in the absence of any commercial or financial relationships that could be construed as a potential conflict of interest.

## Publisher’s note

All claims expressed in this article are solely those of the authors and do not necessarily represent those of their affiliated organizations, or those of the publisher, the editors and the reviewers. Any product that may be evaluated in this article, or claim that may be made by its manufacturer, is not guaranteed or endorsed by the publisher.

## Footnotes

^1^ https://github.com/rrwick/Porechop

^2^ https://github.com/tseemann/abricate

^3^ https://webarchive.nationalarchives.gov.uk/ukgwa/20190501130700/http://bioinformatics.phe.org.uk/legionella/legionella_sbt/php/sbt_homepage.php
