# Supplementary material for: Genomic characterization and assessment of pathogenic potential of Legionella spp. isolates from environmental monitoring
Source: Front Microbiol. 2023 Jan 12;13:1091964. doi: 10.3389/fmicb.2022.1091964 (PMC9879626; doi:10.3389/fmicb.2022.1091964)
Supplement: Supplementary file 1 [file Table_1.DOCX]

**Table S1**: Type strains assemblies used for genomic relatedness analysis. The assemblies were downloaded from the NCBI and crosschecked with

| **Organism Scientific Name** | **Organism Qualifier** | **Taxonomy id** | **Assembly Accession** | **Level** |
| --- | --- | --- | --- | --- |
| *Legionella adelaidensis* | strain: 1762-AUS-E | 45056 | GCF_001467055.1 | Scaffold |
| *Legionella anisa* | strain: FDAARGOS_1480 | 28082 | GCF_019930885.1 | Complete Genome |
| *Legionella antarctica* | strain: TUM19329 | 2708020 | GCF_011764505.1 | Complete Genome |
| *Legionella beliardensis* | strain: NCTC13315 | 91822 | GCF_900452395.1 | Contig |
| *Legionella birminghamensis* | strain: NCTC12437 | 28083 | GCF_900452515.1 | Contig |
| *Legionella bozemanae* | strain: WIGA | 447 | GCF_900640135.1 | Contig |
| *Legionella brunensis* | strain: ATCC 43878 | 29422 | GCF_001467025.1 | Scaffold |
| *Legionella busanensis* | strain: NCTC13316 | 190655 | GCF_900461525.1 | Contig |
| *Legionella cherrii* | strain: NCTC11976 | 28084 | GCF_900635815.1 | Complete Genome |
| *Legionella cincinnatiensis* | strain: NCTC12438 | 28085 | GCF_900452415.1 | Contig |
| *Legionella clemsonensis* | strain: CDC-D5610 | 1867846 | GCF_002240035.1 | Complete Genome |
| *Legionella drancourtii LLAP12* | strain: LLAP12 | 658187 | GCF_000162755.2 | Scaffold |
| *Legionella dumoffi* | strain: NY 23 | 648298 | GCF_000236165.1 | Complete Genome |
| *Legionella drozanskii LLAP-1* | strain: LLAP-1 | 1212489 | GCF_900640075.1 | Scaffold |
| *Legionella erythra* | strain: SE-32A-C8 | 448 | GCF_001467615.1 | Scaffold |
| *Legionella fairfieldensis* | strain: 1725-Aus-E | 45064 | GCF_900640125.1 | Scaffold |
| *Legionella fallonii LLAP-10* | strain: LLAP-10 | 1212491 | GCF_000953135.1 | Complete Genome |
| *Legionella feeleii* | strain: NCTC12022 | 453 | GCF_900452475.1 | Contig |
| *Legionella geestiana* | strain: 1308 | 45065 | GCF_004571195.1 | Complete Genome |
| *Legionella gratiana* | strain: NCTC12388 | 45066 | GCF_900452545.1 | Contig |
| *Legionella hackeliae* | strain: ATCC35250 | 449 | GCF_000953655.1 | Complete Genome |
| *Legionella impletisoli* | strain: OA1-1 | 343510 | GCF_900639875.1 | Contig |
| *Legionella israelensis* | strain: Bercovier 4 | 454 | GCF_004571175.1 | Complete Genome |
| *Legionella jamestowniensis* | strain: JA-26-G1-E2 | 455 | GCF_900640205.1 | Scaffold |
| *Legionella jordanis* | strain: NCTC11533 | 456 | GCF_900637635.1 | Complete Genome |
| *Legionella lansingensis* | strain: NCTC12830 | 45067 | GCF_900187355.1 | Complete Genome |
| *Legionella londiniensis* | strain: 1477 | 45068 | GCF_900639885.1 | Contig |
| *Legionella longbeachae* | strain: FDAARGOS_1481 | 450 | GCF_019930685.1 | Complete Genome |
| *Legionella maceachernii* | strain: NCTC11982 | 466 | GCF_900460175.1 | Contig |
| *Legionella massiliensis* | strain: LegA | 1034943 | GCF_000756815.1 | Contig |
| *Legionella moravica* | strain: NCTC12239 | 39962 | GCF_900452715.1 | Contig |
| *Legionella nagasakiensis* | strain: JCM 15315 | 535290 | GCF_900639915.1 | Scaffold |
| *Legionella nautarum* | strain: ATCC 49506 | 45070 | GCF_001467895.1 | Scaffold |
| *Legionella norrlandica* | strain: LEGN | 1498499 | GCF_000770585.1 | Contig |
| *Legionella oakridgensis* | strain: Oak Ridge-10 | 29423 | GCF_001467925.1 | Scaffold |
| *Legionella parisiensis* | strain: NCTC11983 | 45071 | GCF_900461585.1 | Contig |
| *Legionella pneumophila* | strain: FDAARGOS_779 | 446 | GCF_006364635.1 | Complete Genome |
| *Legionella pneumophila subsp. fraseri* | strain: Los Angeles 1 | 91892 | GCF_900639965.1 | Scaffold |
| *Legionella pneumophila subsp. pascullei* | strain: NCTC12273 | 91890 | GCF_900637585.1 | Complete Genome |
| *Legionella pneumophila subsp. pneumophila* | strain: FDAARGOS_202 | 91891 | GCF_002082955.2 | Contig |
| *Legionella qingyii* | strain: km488 | 2184757 | GCF_003184185.1 | Scaffold |
| *Legionella quateirensis* | strain: NCTC12376 | 45072 | GCF_900452695.1 | Contig |
| *Legionella quinlivanii* | strain: CDC#1442-AUS-E | 45073 | GCF_001467975.1 | Scaffold |
| *Legionella rubrilucens* | strain: WA-270A-C2 | 458 | GCF_900640015.1 | Contig |
| *Legionella sainthelensi* | strain: NCTC11988 | 28087 | GCF_900637685.1 | Complete Genome |
| *Legionella santicrucis* | strain: SC-63-C7 | 45074 | GCF_001468135.1 | Scaffold |
| *Legionella saoudiensis* | strain: LS-1 | 1750561 | GCF_001465875.1 | Scaffold |
| *Legionella septentrionalis* | strain: km711 | 2498109 | GCF_003989745.1 | Contig |
| *Legionella shakespearei DSM 23087* | strain: ATCC 49655 | 1122169 | GCF_001468025.1 | Scaffold |
| *Legionella spiritensis* | strain: NCTC11990 | 452 | GCF_900186965.1 | Complete Genome |
| *Legionella steelei* | strain: IMVS3376 | 947033 | GCF_001468005.1 | Scaffold |
| *Legionella steigerwaltii* | strain: NCTC11991 | 460 | GCF_900452835.1 | Contig |
| *Legionella taurinensis* | strain: NCTC13314 | 70611 | GCF_900452865.1 | Contig |
| *Legionella tucsonensis* | strain: ATCC 49180 | 40335 | GCF_001468035.1 | Scaffold |
| *Legionella tunisiensis* | strain: LegM | 1034944 | GCF_000308315.1 | Contig |
| *Legionella wadsworthii* | strain: NCTC11532 | 28088 | GCF_900452925.1 | Contig |
| *Legionella waltersii* | strain: NCTC13017 | 66969 | GCF_900187095.1 | Complete Genome |
| *Legionella worsleiensis* | strain: NCTC12377 | 45076 | GCF_900453045.1 | Contig |
| *Legionella yabuuchiae* | strain: OA1-2 | 376727 | GCF_900640115.1 | Contig |

**Table S2**: General characteristics of the de novo assembly of the Legionella strains sequenced in this study. The assemblies were conducted by Unicycler and functionally annotated by Prokka

|  | **QUAST** | | | | | | | | | **PROKKA** | | | | | |
| --- | --- | --- | --- | --- | --- | --- | --- | --- | --- | --- | --- | --- | --- | --- | --- |
| **Sample ID** | **Largest contig** | **Total length** | **GC (%)** | **N50** | **N75** | **L50** | **L75** | **# N's per 100 kbp** | **bases** | **CDS** | **contigs** | **repeat_region** | **rRNA** | **tmRNA** | **tRNA** |
| PATHC001 | 3511425 | 3655504 | 39.64 | 3511425 | 3511425 | 1 | 1 | 0 | 3655504 | 3215 | 2 |  | 9 | 1 | 43 |
| PATHC010 | 3464227 | 3551678 | 38.37 | 3464227 | 3464227 | 1 | 1 | 0 | 3551678 | 3168 | 2 |  | 9 | 1 | 43 |
| PATHC012 | 3448976 | 3524552 | 38.37 | 3448976 | 3448976 | 1 | 1 | 0 | 3524552 | 3142 | 2 |  | 9 | 1 | 43 |
| PATHC013 | 3811972 | 3879210 | 38.15 | 3811972 | 3811972 | 1 | 1 | 0 | 3879210 | 3369 | 3 | 1 | 12 | 1 | 44 |
| PATHC014 | 3425525 | 3584768 | 38.36 | 3425525 | 3425525 | 1 | 1 | 0 | 3584768 | 3196 | 3 |  | 9 | 1 | 43 |
| PATHC015 | 3445762 | 3916342 | 38.32 | 3445762 | 3445762 | 1 | 1 | 0 | 3920518 | 3533 | 25 | 1 | 9 | 1 | 43 |
| PATHC017 | 3627098 | 3844790 | 39.65 | 3627098 | 3627098 | 1 | 1 | 0 | 3844790 | 3411 | 3 |  | 9 | 1 | 43 |
| PATHC018 | 3505984 | 3593849 | 38.42 | 3505984 | 3505984 | 1 | 1 | 0 | 3593849 | 3215 | 2 | 1 | 9 | 1 | 43 |
| PATHC019 | 3647042 | 3797474 | 39.68 | 3647042 | 3647042 | 1 | 1 | 0 | 3797474 | 3363 | 2 |  | 9 | 1 | 43 |
| PATHC002 | 3469552 | 3469552 | 38.36 | 3469552 | 3469552 | 1 | 1 | 0 | 3469552 | 3108 | 1 | 1 | 9 | 1 | 43 |
| PATHC020 | 3501725 | 3604270 | 42.98 | 3501725 | 3501725 | 1 | 1 | 0 | 3605084 | 3069 | 8 |  | 9 | 1 | 42 |
| PATHC021 | 3332865 | 3332865 | 38.28 | 3332865 | 3332865 | 1 | 1 | 0 | 3332865 | 2934 | 1 |  | 9 | 1 | 43 |
| PATHC022 | 3511780 | 3587818 | 38.39 | 3511780 | 3511780 | 1 | 1 | 0 | 3587818 | 3182 | 4 | 1 | 9 | 1 | 43 |
| PATHC023 | 4267150 | 4464645 | 38.25 | 4267150 | 4267150 | 1 | 1 | 0 | 4464645 | 3977 | 3 |  | 9 | 1 | 43 |
| PATHC024 | 3381543 | 3457119 | 38.36 | 3381543 | 3381543 | 1 | 1 | 0 | 3457119 | 3065 | 2 |  | 9 | 1 | 43 |
| PATHC025 | 3471815 | 3471815 | 38.35 | 3471815 | 3471815 | 1 | 1 | 0 | 3471815 | 3062 | 1 |  | 9 | 1 | 43 |
| PATHC026 | 3789434 | 3840392 | 38.25 | 3789434 | 3789434 | 1 | 1 | 0 | 3840392 | 3335 | 2 |  | 12 | 1 | 44 |
| PATHC027 | 3461893 | 3461893 | 38.35 | 3461893 | 3461893 | 1 | 1 | 0 | 3461893 | 3059 | 1 |  | 9 | 1 | 43 |
| PATHC028 | 3457405 | 3462032 | 38.35 | 3457405 | 3457405 | 1 | 1 | 0 | 3462366 | 3063 | 3 |  | 9 | 1 | 43 |
| PATHC029 | 2529056 | 4486832 | 38.24 | 2529056 | 980083 | 1 | 2 | 0 | 4486832 | 3996 | 9 |  | 9 | 1 | 43 |
| PATHC030 | 3359777 | 3359777 | 38.33 | 3359777 | 3359777 | 1 | 1 | 0 | 3359777 | 2974 | 1 |  | 9 | 1 | 43 |
| PATHC003 | 3526734 | 3694195 | 38.33 | 3526734 | 3526734 | 1 | 1 | 0 | 3694195 | 3305 | 4 | 1 | 9 | 1 | 43 |
| PATHC030 | 3513681 | 3601132 | 38.23 | 3513681 | 3513681 | 1 | 1 | 0 | 3601132 | 3198 | 2 | 1 | 9 | 1 | 43 |
| PATHC031 | 3379205 | 3448333 | 38.03 | 3379205 | 3379205 | 1 | 1 | 0 | 3448333 | 3043 | 2 | 1 | 9 | 1 | 43 |
| PATHC032 | 3623374 | 3846133 | 38.24 | 3623374 | 3623374 | 1 | 1 | 0 | 3846560 | 3468 | 5 | 1 | 9 | 1 | 43 |
| PATHC033 | 3400965 | 3400965 | 38.32 | 3400965 | 3400965 | 1 | 1 | 0 | 3400965 | 3018 | 1 |  | 9 | 1 | 43 |
| PATHC034 | 3699289 | 3895788 | 39.43 | 3699289 | 3699289 | 1 | 1 | 0 | 3895788 | 3361 | 2 |  | 9 | 1 | 43 |
| PATHC035 | 3599626 | 3839455 | 39.7 | 3599626 | 3599626 | 1 | 1 | 0 | 3839455 | 3393 | 5 |  | 9 | 1 | 43 |
| PATHC036 | 3389513 | 3470186 | 38.27 | 3389513 | 3389513 | 1 | 1 | 0 | 3470186 | 3079 | 2 |  | 9 | 1 | 43 |
| PATHC037 | 442404 | 4272683 | 38.83 | 235662 | 138061 | 7 | 13 | 0 | 4276216 | 3851 | 64 |  | 6 | 1 | 43 |
| PATHC038 | 3341950 | 3516567 | 38.13 | 3341950 | 3341950 | 1 | 1 | 0 | 3517029 | 3170 | 3 |  | 9 | 1 | 43 |
| PATHC004 | 3345338 | 3418913 | 38.28 | 3345338 | 3345338 | 1 | 1 | 0 | 3418913 | 3027 | 2 |  | 9 | 1 | 43 |
| PATHC040 | 2347655 | 3761786 | 39.66 | 2347655 | 1237030 | 1 | 2 | 0 | 3762120 | 3335 | 10 |  | 9 | 1 | 43 |
| PATHC041 | 3396402 | 3528987 | 38.2 | 3396402 | 3396402 | 1 | 1 | 0 | 3528987 | 3155 | 2 | 1 | 9 | 1 | 43 |
| PATHC042 | 3369699 | 3442535 | 38.34 | 3369699 | 3369699 | 1 | 1 | 0 | 3442535 | 3059 | 2 |  | 9 | 1 | 43 |
| PATHC005 | 3396996 | 3604558 | 38.32 | 3396996 | 3396996 | 1 | 1 | 0 | 3604558 | 3221 | 3 |  | 9 | 1 | 43 |
| PATHC006 | 3462052 | 3679366 | 38.31 | 3462052 | 3462052 | 1 | 1 | 0 | 3679366 | 3273 | 3 |  | 9 | 1 | 43 |
| PATHC007 | 3409121 | 3409121 | 38.3 | 3409121 | 3409121 | 1 | 1 | 0 | 3409121 | 3007 | 1 | 1 | 9 | 1 | 43 |
| PATHC009 | 3710389 | 3938292 | 39.67 | 3710389 | 3710389 | 1 | 1 | 0 | 3938292 | 3490 | 2 |  | 9 | 1 | 43 |

**Table S3**: Contigs which were circular (closed) as per Unicycler results were blasted against NCBI nr database. The table contains BLASTn results together with the relevant scores. In some isolates there are more than one plasmid, while there are samples where no plasmid was

|  | PLASMID 1 | | | PLASMID 2 | | |
| --- | --- | --- | --- | --- | --- | --- |
| Sample ID | query sequence | Description | Max Score | query sequence | Description | Max Score |
| PATHC001 | contig 2 | *Legionella pneumophila* strain L10-023 plasmid, complete sequence | 232900 |  |  |  |
| PATHC010 | contig 2 | *Legionella pneumophila* subsp. pneumophila strain FFI329 plasmid, complete sequence | 65928 |  |  |  |
| PATHC012 | contig 2 | *Legionella pneumophila* strain C9_S plasmid unamed2, complete sequence | 74354 |  |  |  |
| PATHC013 | contig 2 | *Legionella longbeachae* strain B1445CHC plasmid pB1445CHC_73K, complete sequence | 63261 | contig 3 | Cloning vector pNT562 DNA, complete sequence | 278 |
| PATHC014 | contig 2 | *Legionella pneumophila* subsp. pneumophila strain FFI329 plasmid, complete sequence | 65928 | contig 3 | *Legionella pneumophila* strain D-4040 plasmid unnamed2, complete sequence | 113000 |
| PATHC015 |  |  |  |  |  |  |
| PATHC017 | contig 3 | *Legionella pneumophila* strain ERS1305867 plasmid unnamed, complete sequence | 99169 | contig 3 | *Legionella pneumophila* subsp. pneumophila strain FFI329 plasmid, complete sequence | 65928 |
| PATHC018 | contig 2 | *Legionella pneumophila* subsp. pneumophila strain FFI329 plasmid, complete sequence | 65928 |  |  |  |
| PATHC019 | contig 2 | *Legionella sainthelensi* strain LA01-117 plasmid pLA01-117_150k, complete sequence | 253000 |  |  |  |
| PATHC002 |  |  |  |  |  |  |
| PATHC020 | contig 2 | *Legionella pneumophila* subsp. pneumophila strain Birmingham 1 (D-7470) chromosome, complete genome | 1520 |  |  |  |
| PATHC021 |  |  |  |  |  |  |
| PATHC022 | contig 2 | *Legionella pneumophila* subsp. pneumophila LPE509 plasmid, complete sequence | 77848 |  |  |  |
| PATHC023 | contig 2 | *Legionella anisa* isolate UMCG_3A plasmid p3A1, complete sequence | 175000 | contig 3 | *Legionella anisa* isolate UMCG_3A plasmid p3A2, complete sequence | 76105 |
| PATHC024 | contig 2 | *Legionella pneumophila* subsp. fraseri strain D-4058 plasmid unnamed, complete sequence | 63725 |  |  |  |
| PATHC025 |  |  |  |  |  |  |
| PATHC026 | contig 2 | *Legionella anisa* isolate UMCG_3A plasmid p3A2, complete sequence | 19001 |  |  |  |
| PATHC027 |  |  |  |  |  |  |
| PATHC028 |  |  |  |  |  |  |
| PATHC029 | contig 5 | *Legionella anisa* isolate UMCG_3A plasmid p3A2, complete sequence | 87006 |  |  |  |
| PATHC030 | contig 2 | *Legionella pneumophila* subsp. pneumophila strain Flint 2 (D-7477) plasmid unnamed, complete sequence | 228000 |  |  |  |
| PATHC003 |  |  |  |  |  |  |
| PATHC030 | contig 2 | *Legionella pneumophila* subsp. pneumophila strain FFI329 plasmid, complete sequence | 65928 |  |  |  |
| PATHC031 | contig 2 | *Legionella pneumophila* str. Lens plasmid pLPL, complete sequence | 55376 |  |  |  |
| PATHC032 | contig 2 | *Legionella pneumophila* strain ERS1305867 plasmid unnamed, complete sequence | 83493 | contig 3 | *Legionella pneumophila* str. Lens plasmid pLPL, complete sequence | 64116 |
| PATHC033 |  |  |  |  |  |  |
| PATHC034 | contig 2 | *Legionella longbeachae* strain B3526CHC plasmid pB3526CHC_150k, complete sequence | 62680 |  |  |  |
| PATHC035 | contig 2 | *Legionella pneumophila* subsp. pneumophila strain Allentown 1 (D-7475) plasmid unnamed1, complete sequence | 239000 | contig 3 | *Legionella longbeachae* strain F1157CHC chromosome, complete genome | 8569 |
| PATHC036 | contig 2 | *Legionella pneumophila* str. Lens plasmid pLPL, complete sequence | 48078 |  |  |  |
| PATHC037 | contig 20 | *Legionella anisa* isolate UMCG_3A plasmid p3A2, complete sequence | 15396 |  |  |  |
| PATHC038 | contig 2 | Legionella sp. MW5194 plasmid unnamed1, complete sequence | 87755 |  |  |  |
| PATHC004 | contig 2 | *Legionella pneumophila* strain E5_N plasmid unnamed1, complete sequence | 98318 |  |  |  |
| PATHC040 | contig 2 | *Legionella pneumophila* strain L10-023 plasmid, complete sequence | 118000 |  |  |  |
| PATHC041 | contig 2 | *Legionella pneumophila* strain ERS1305867 plasmid unnamed, complete sequence | 69913 |  |  |  |
| PATHC042 | contig 2 | *Legionella pneumophila* strain E5_N plasmid unnamed1, complete sequence | 74912 |  |  |  |
| PATHC005 | contig 2 | *Legionella pneumophila* strain ERS1305867 plasmid unnamed, complete sequence | 73161 | contig 3 | *Legionella pneumophila* str. Lens plasmid pLPL, complete sequence | 45993 |
| PATHC006 | contig 2 | *Legionella pneumophila* subsp. pneumophila strain Flint 2 (D-7477) plasmid unnamed, complete sequence | 132000 | contig 3 | *Legionella pneumophila* str. Lens plasmid pLPL, complete sequence | 49724 |
| PATHC007 |  |  |  |  |  |  |
| PATHC009 | Contig 2 | *Legionella longbeachae* strain B1445CHC plasmid pB1445CHC_150k, complete sequence | 5840 |  |  |  |
| PATHC026 |  |  |  |  |  |  |

**
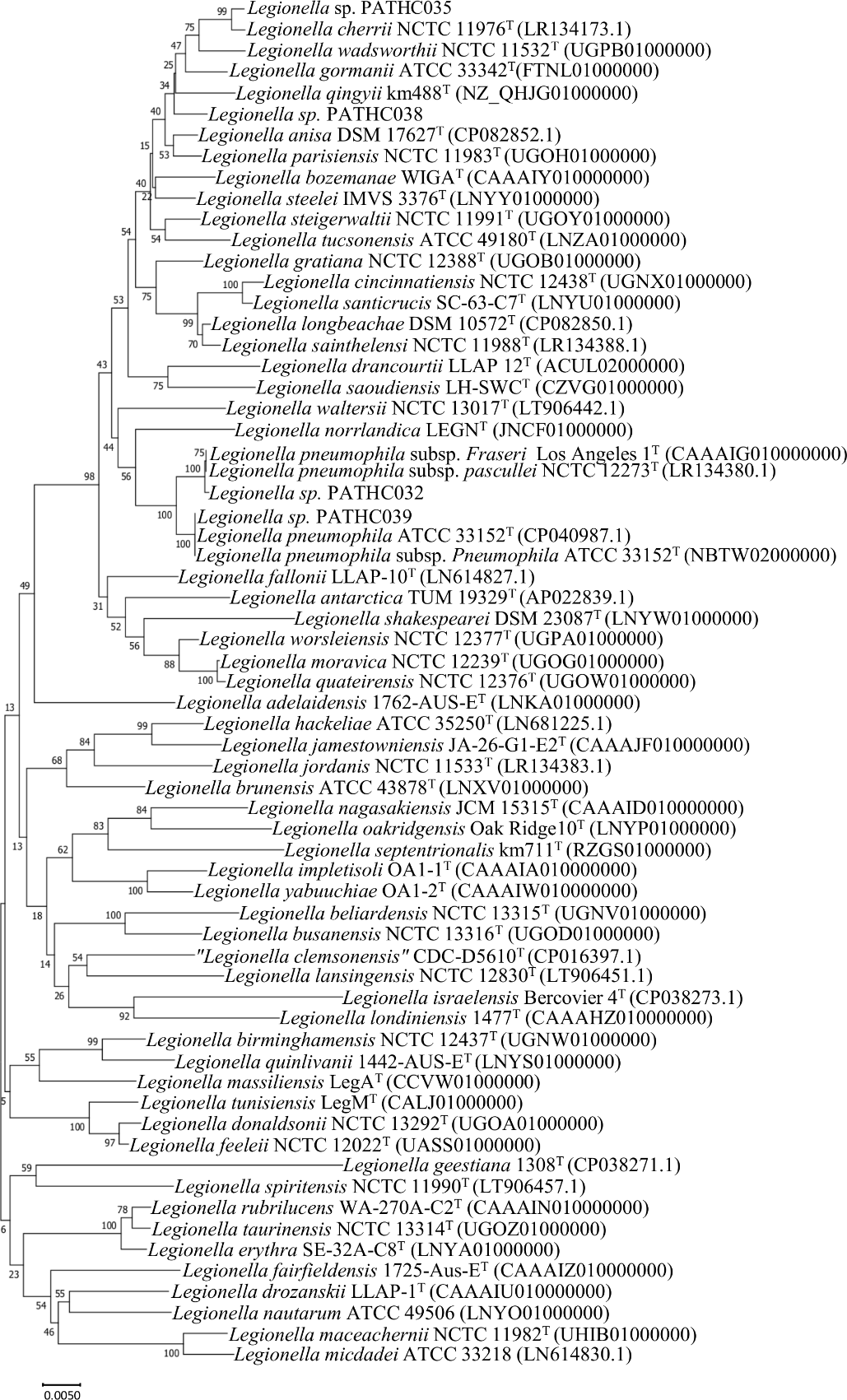
**

**Figure S1:** 16S rRNA tree constructed based on 1423 nucleotide positions of the gene including 16S rRNA genes of Legionella type strains and isolates from this study PATHCO032, PATHC035, PATHC038 and PATHC039 suspected to be novel species.

**Pangenome analysis of non-pneumophilla isolates sequenced in this study and obtained from NCBI**

***Legionella anisa***


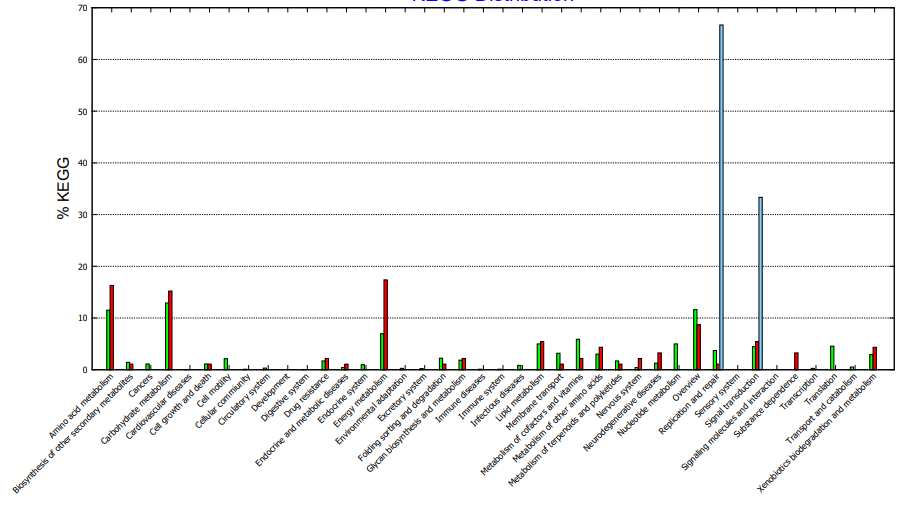

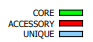


**Figure S2**: Functional distribution of Legionella anisa core, accessory and unique as determined by genes Kyoto Encyclopedia of Genes (Genomes (KEGG) pathway analysis embeded in Bacterial Pan Genome Analysis Pipeline (BPGA). Strains included in the pangenome analysis include 2 isolates sequenced in this study and 6 genomes downloaded from NCBI


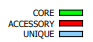

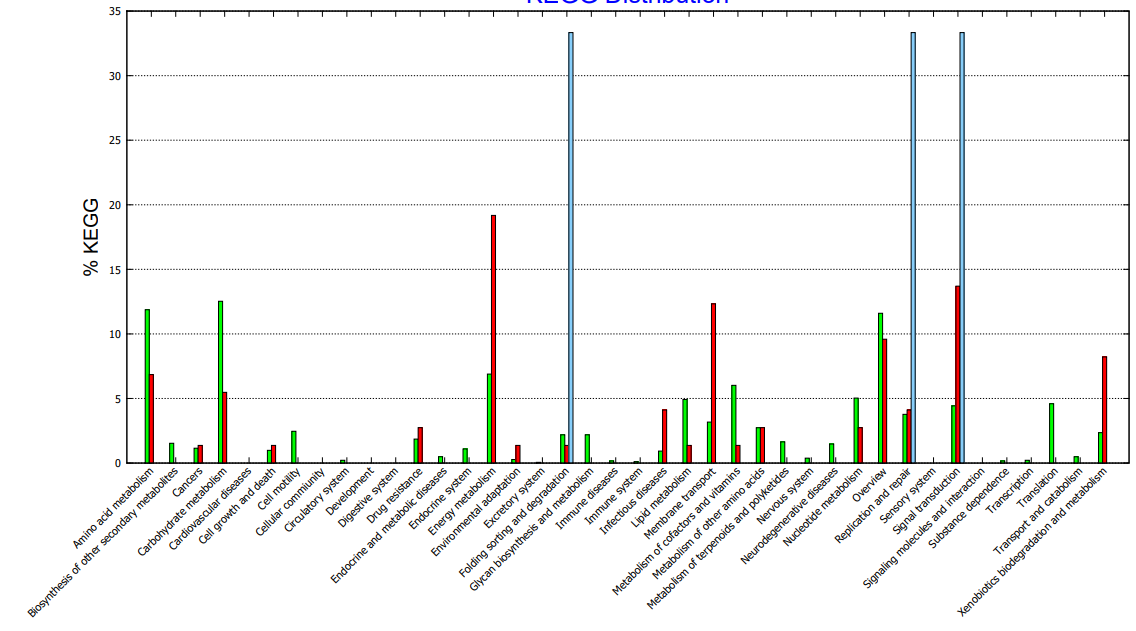


***Legionella dumoffii***

**Figure S3**: Functional distribution of Legionella dumoffii core, accessory and unique as determined by genes Kyoto Encyclopedia of Genes (Genomes (KEGG) pathway analysis embeded in Bacterial Pan Genome Analysis Pipeline (BPGA). Strains included in the pangenome analysis include 6 isolates sequenced in this study and 2 genomes downloaded from NCBI


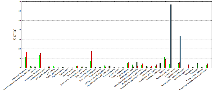

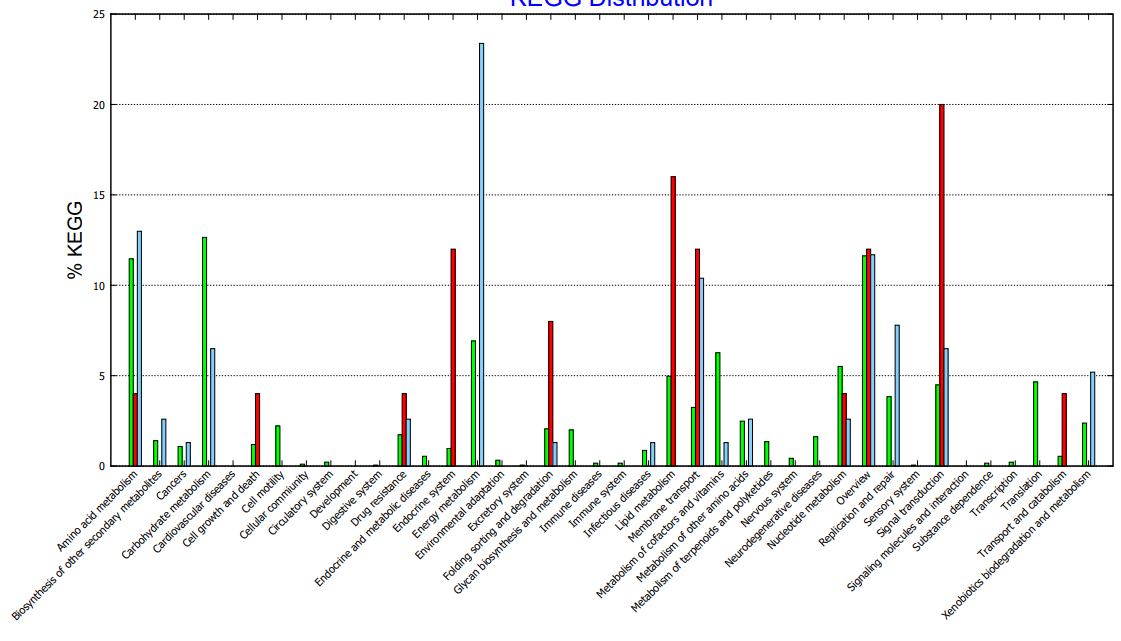


***Legionella gormanii***

**Figure S4**: Functional distribution of Legionella gormanii core, accessory and unique as determined by genes Kyoto Encyclopedia of Genes (Genomes (KEGG) pathway analysis embeded in Bacterial Pan Genome Analysis Pipeline (BPGA). Strains included in the pangenome analysis include 2 isolates sequenced in this study and 2 genomes downloaded from NCBI


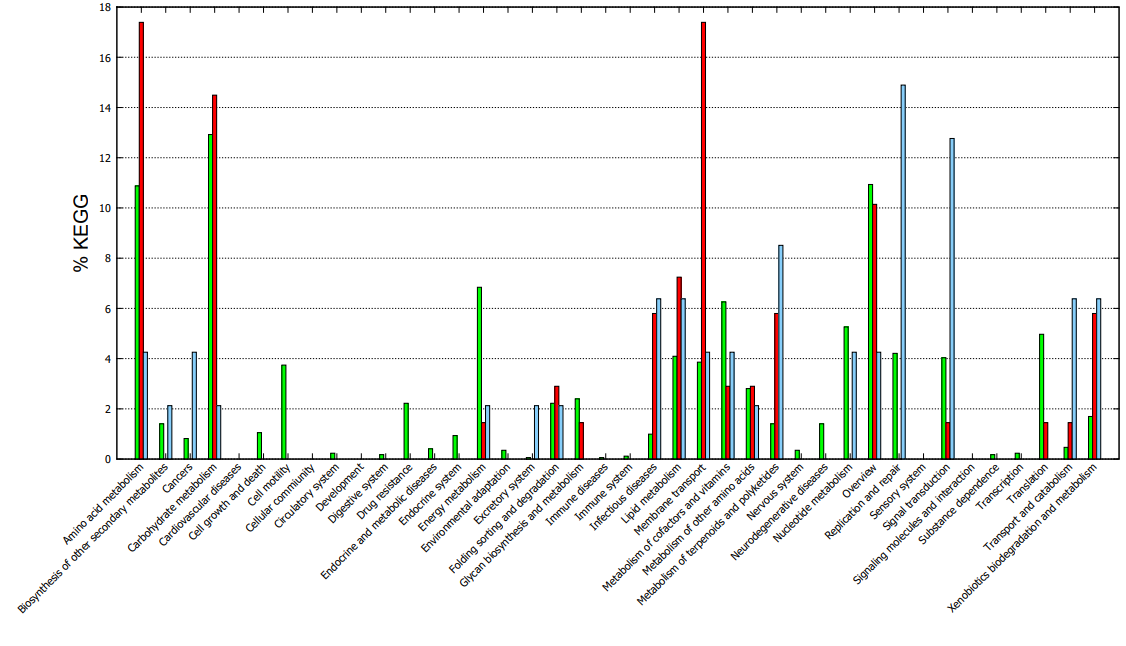


***Legionella*** ***quinlivanii***

**Figure S5:** Functional distribution of Legionella quinlivanii core, accessory and unique as determined by genes Kyoto Encyclopedia of Genes (Genomes (KEGG) pathway analysis embeded in Bacterial Pan Genome Analysis Pipeline (BPGA). Strains included in the pangenome analysis include 1 isolate sequenced in this study and 2 genomes downloaded from NCBI


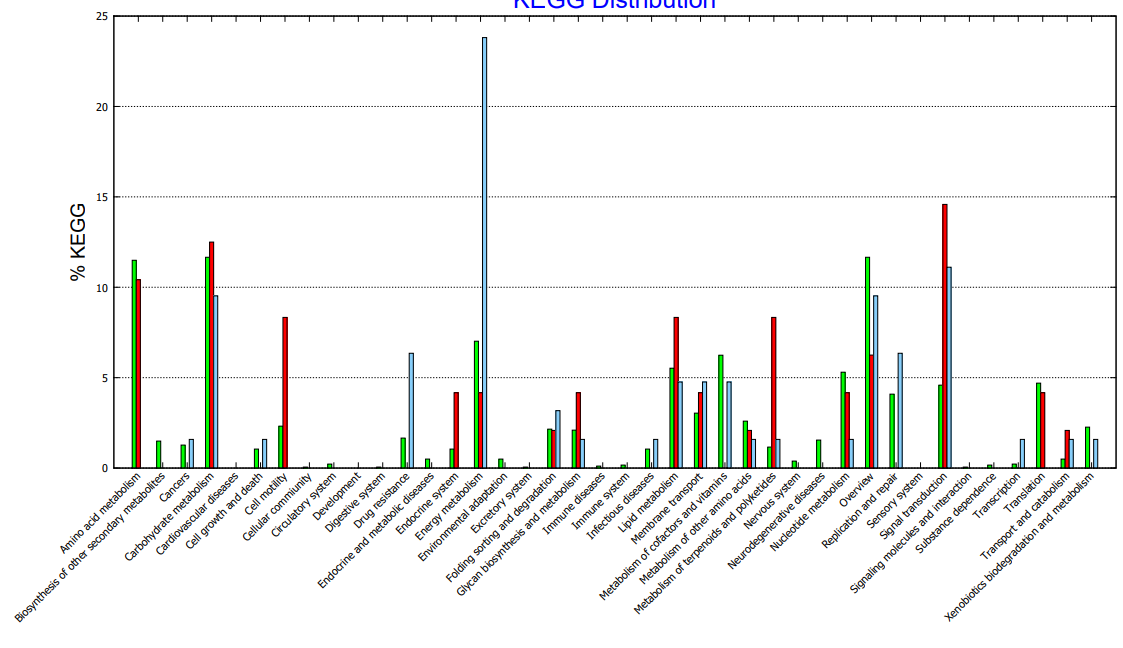


***Legionella cherrii***

**Figure S6:** Functional distribution of Legionella cherrii core, accessory and unique as determined by genes Kyoto Encyclopedia of Genes (Genomes (KEGG) pathway analysis embeded in Bacterial Pan Genome Analysis Pipeline (BPGA). Strains included in the pangenome analysis include 2 isolates sequenced in this study and 1 genome downloaded from NCBI

**Table S4:** Results of the pangenome analysis of *Legionella pneumophila* strains including the strains sequenced in this study and complete genomes downloaded from the NCBI**.** The number of core, accessory, unique and exclusively absent genes was determined by Bacterial Pan Genome Analysis Pipeline (BPGA).

| **Genome no.** | **Organism name** | **No. of core genes** | **No. of accessory genes** | **No. of unique genes** | **No. of exclusively absent genes** |
| --- | --- | --- | --- | --- | --- |
| 1 | PATHC02 | 1938 | 910 | 42 | 0 |
| 2 | PATHC03 | 1938 | 950 | 0 | 0 |
| 3 | PATHC04 | 1938 | 988 | 0 | 0 |
| 4 | PATHC05 | 1938 | 1117 | 1 | 1 |
| 5 | PATHC06 | 1938 | 1182 | 4 | 0 |
| 6 | PATHC07 | 1938 | 969 | 0 | 0 |
| 7 | PATHC010 | 1938 | 1109 | 4 | 0 |
| 8 | PATHC012 | 1938 | 1057 | 0 | 0 |
| 9 | PATHC014 | 1938 | 1124 | 0 | 0 |
| 10 | PATHC015 | 1938 | 1312 | 1 | 0 |
| 11 | PATHC018 | 1938 | 1070 | 54 | 0 |
| 12 | PATHC021 | 1938 | 923 | 0 | 0 |
| 13 | PATHC022 | 1938 | 1097 | 0 | 0 |
| 14 | PATHC024 | 1938 | 1026 | 0 | 0 |
| 15 | PATHC025 | 1938 | 1015 | 0 | 0 |
| 16 | PATHC027 | 1938 | 1015 | 0 | 0 |
| 17 | PATHC028 | 1938 | 1018 | 1 | 0 |
| 18 | PATHC030 | 1938 | 1203 | 7 | 0 |
| 19 | PATHC031 | 1938 | 1128 | 0 | 0 |
| 20 | PATHC032 | 1938 | 966 | 48 | 16 |
| 21 | PATHC033 | 1938 | 1283 | 36 | 0 |
| 22 | PATHC034 | 1938 | 976 | 0 | 0 |
| 23 | PATHC037 | 1938 | 1044 | 23 | 0 |
| 24 | PATHC039 | 1938 | 997 | 104 | 14 |
| 25 | PATHC041 | 1938 | 1086 | 0 | 0 |
| 26 | PATHC042 | 1938 | 1025 | 0 | 0 |
| 27 | Legionella pneumophila 230099 Alcoy | 1938 | 1052 | 8 | 1 |
| 28 | 80-045 | 1938 | 964 | 12 | 0 |
| 29 | 11052018-5 | 1938 | 972 | 2 | 0 |
| 30 | Albuquerque1(D-7474) | 1938 | 1138 | 14 | 1 |
| 31 | Allentown1(D-7475) | 1938 | 1173 | 26 | 0 |
| 32 | ATCC43290 | 1938 | 963 | 0 | 0 |
| 33 | AUSMDU00010536 | 1938 | 998 | 0 | 0 |
| 34 | AW-13-4 | 1938 | 1176 | 0 | 0 |
| 35 | Bellingham1(D-7473) | 1938 | 958 | 2 | 0 |
| 36 | Birmingham1(D-7470) | 1938 | 977 | 15 | 1 |
| 37 | Burlington1(D-7841) | 1938 | 975 | 0 | 0 |
| 38 | C1S | 1938 | 999 | 6 | 0 |
| 39 | C2S | 1938 | 975 | 0 | 0 |
| 40 | C3O | 1938 | 1026 | 0 | 0 |
| 41 | C4S | 1938 | 990 | 1 | 0 |
| 42 | C5P | 1938 | 991 | 0 | 0 |
| 43 | C6S | 1938 | 977 | 0 | 0 |
| 44 | C7O | 1938 | 945 | 0 | 0 |
| 45 | C8S | 1938 | 1043 | 0 | 0 |
| 46 | C9S | 1938 | 1066 | 9 | 0 |
| 47 | C10S | 1938 | 944 | 0 | 0 |
| 48 | C11O | 1938 | 990 | 0 | 0 |
| 49 | Corby | 1938 | 1061 | 10 | 0 |
| 50 | D-3137 | 1938 | 1036 | 0 | 0 |
| 51 | D-4040 | 1938 | 1188 | 6 | 0 |
| 52 | D-4058 | 1938 | 1157 | 0 | 0 |
| 53 | D-4954 | 1938 | 1079 | 40 | 0 |
| 54 | D-5265 | 1938 | 1121 | 3 | 0 |
| 55 | D-5387 | 1938 | 1034 | 0 | 0 |
| 56 | D-5744 | 1938 | 1046 | 1 | 0 |
| 57 | D5945 | 1938 | 1035 | 0 | 0 |
| 58 | D6026 | 1938 | 1034 | 0 | 0 |
| 59 | D-7119 | 1938 | 969 | 1 | 0 |
| 60 | D-7158 | 1938 | 1037 | 0 | 0 |
| 61 | D-7630 | 1938 | 994 | 6 | 0 |
| 62 | D-7631 | 1938 | 995 | 0 | 0 |
| 63 | D-7632 | 1938 | 993 | 0 | 0 |
| 64 | D-7708 | 1938 | 938 | 37 | 2 |
| 65 | D-7787 | 1938 | 982 | 23 | 0 |
| 66 | Dallas1E | 1938 | 1057 | 0 | 0 |
| 67 | Detroit-1 | 1938 | 1026 | 1 | 0 |
| 68 | E1P | 1938 | 986 | 0 | 0 |
| 69 | E2N | 1938 | 1012 | 2 | 0 |
| 70 | E3N | 1938 | 1013 | 8 | 0 |
| 71 | E4N | 1938 | 987 | 0 | 0 |
| 72 | E5N | 1938 | 1058 | 0 | 0 |
| 73 | E6N | 1938 | 978 | 0 | 0 |
| 74 | E7O | 1938 | 991 | 0 | 0 |
| 75 | E8O | 1938 | 1028 | 0 | 0 |
| 76 | E9O | 1938 | 982 | 14 | 0 |
| 77 | E10P | 1938 | 989 | 0 | 0 |
| 78 | E11U | 1938 | 980 | 0 | 0 |
| 79 | ERS1305867 | 1938 | 1044 | 30 | 0 |
| 80 | F-4185 | 1938 | 967 | 0 | 0 |
| 81 | F-4198 | 1938 | 1080 | 28 | 0 |
| 82 | F4468 | 1938 | 994 | 0 | 0 |
| 83 | F4469 | 1938 | 996 | 0 | 0 |
| 84 | FDAARGOS779 | 1938 | 986 | 0 | 0 |
| 85 | FDAARGOS1482 | 1938 | 1001 | 4 | 0 |
| 86 | FDAARGOS1483 | 1938 | 988 | 1 | 0 |
| 87 | FFI102 | 1938 | 978 | 0 | 0 |
| 88 | FFI103 | 1938 | 981 | 1 | 0 |
| 89 | FFI104 | 1938 | 952 | 0 | 0 |
| 90 | FFI105 | 1938 | 950 | 0 | 0 |
| 91 | FFI329 | 1938 | 979 | 1 | 0 |
| 92 | FFI337 | 1938 | 952 | 0 | 0 |
| 93 | Flint2(D-7477) | 1938 | 1215 | 0 | 0 |
| 94 | HL06041035 | 1938 | 972 | 29 | 2 |
| 95 | Knoxville1(D-7468) | 1938 | 974 | 0 | 0 |
| 96 | L10-023 | 1938 | 1218 | 21 | 1 |
| 97 | Lansing3 | 1938 | 1043 | 54 | 0 |
| 98 | Lens | 1938 | 959 | 1 | 0 |
| 99 | Lorraine | 1938 | 1100 | 5 | 0 |
| 100 | LosAngeles1(D-7696) | 1938 | 1033 | 10 | 0 |
| 101 | Lp02substr.Hextuple2q | 1938 | 433 | 0 | 0 |
| 102 | Lp02substr.hextuple3a | 1938 | 434 | 0 | 0 |
| 103 | LPE509 | 1938 | 1049 | 3 | 0 |
| 104 | NCTC11193 | 1938 | 1006 | 0 | 0 |
| 105 | NCTC11286 | 1938 | 972 | 0 | 0 |
| 106 | NCTC11404 | 1938 | 958 | 0 | 0 |
| 107 | NCTC11985 | 1938 | 981 | 20 | 0 |
| 108 | NCTC12179 | 1938 | 1042 | 30 | 3 |
| 109 | NCTC12180 | 1938 | 963 | 0 | 0 |
| 110 | NCTC12272 | 1938 | 1037 | 0 | 0 |
| 111 | NCTC12273 | 1938 | 1041 | 0 | 0 |
| 112 | NY23(D-7705) | 1938 | 1050 | 3 | 0 |
| 113 | NY24(D-7706) | 1938 | 1052 | 0 | 0 |
| 114 | OLDA | 1938 | 1154 | 0 | 0 |
| 115 | Paris | 1938 | 1157 | 0 | 0 |
| 116 | PartS-Lpneumophila-RM8376 | 1938 | 988 | 0 | 0 |
| 117 | Philadelphia1ATCC | 1938 | 1005 | 0 | 0 |
| 118 | Philadelphia1CDC | 1938 | 988 | 0 | 0 |
| 119 | Philadelphia2 | 1938 | 1006 | 0 | 0 |
| 120 | Philadelphia3 | 1938 | 982 | 0 | 0 |
| 121 | Philadelphia4 | 1938 | 1005 | 0 | 0 |
| 122 | Philadelphia1 | 1938 | 987 | 0 | 0 |
| 123 | Philadelphia-1 | 1938 | 987 | 0 | 0 |
| 124 | Pontiac | 1938 | 1065 | 8 | 0 |
| 125 | ST23 | 1938 | 1035 | 26 | 0 |
| 126 | ST37 | 1938 | 1019 | 2 | 0 |
| 127 | ST42 | 1938 | 970 | 14 | 0 |
| 128 | ST62 | 1938 | 1059 | 4 | 0 |
| 129 | ThunderBay | 1938 | 1043 | 1 | 0 |
| 130 | Toronto-2005 | 1938 | 1023 | 48 | 0 |
| 131 | U8W(D-7160) | 1938 | 1040 | 1 | 0 |
| 132 | Lpm7613 | 1938 | 989 | 0 | 0 |

**
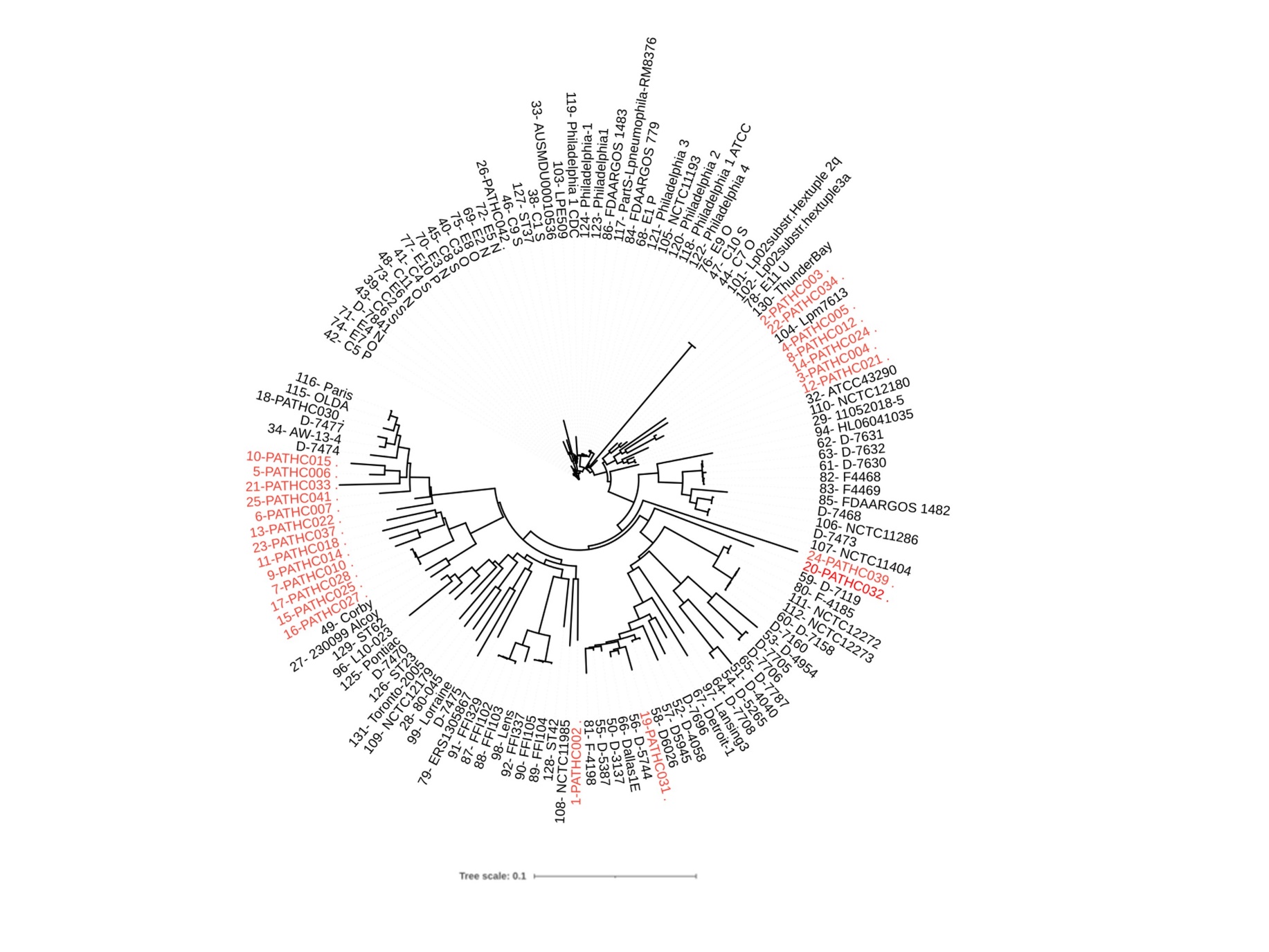
**

**Figure S7:** Pangenome tree of *Legionella pneumophila* strains including the strains sequenced in this study and complete genomes downloaded from the NCBI. The pangenome trees were constructed with Bacterial Pan Genome Analysis Pipeline (BPGA).


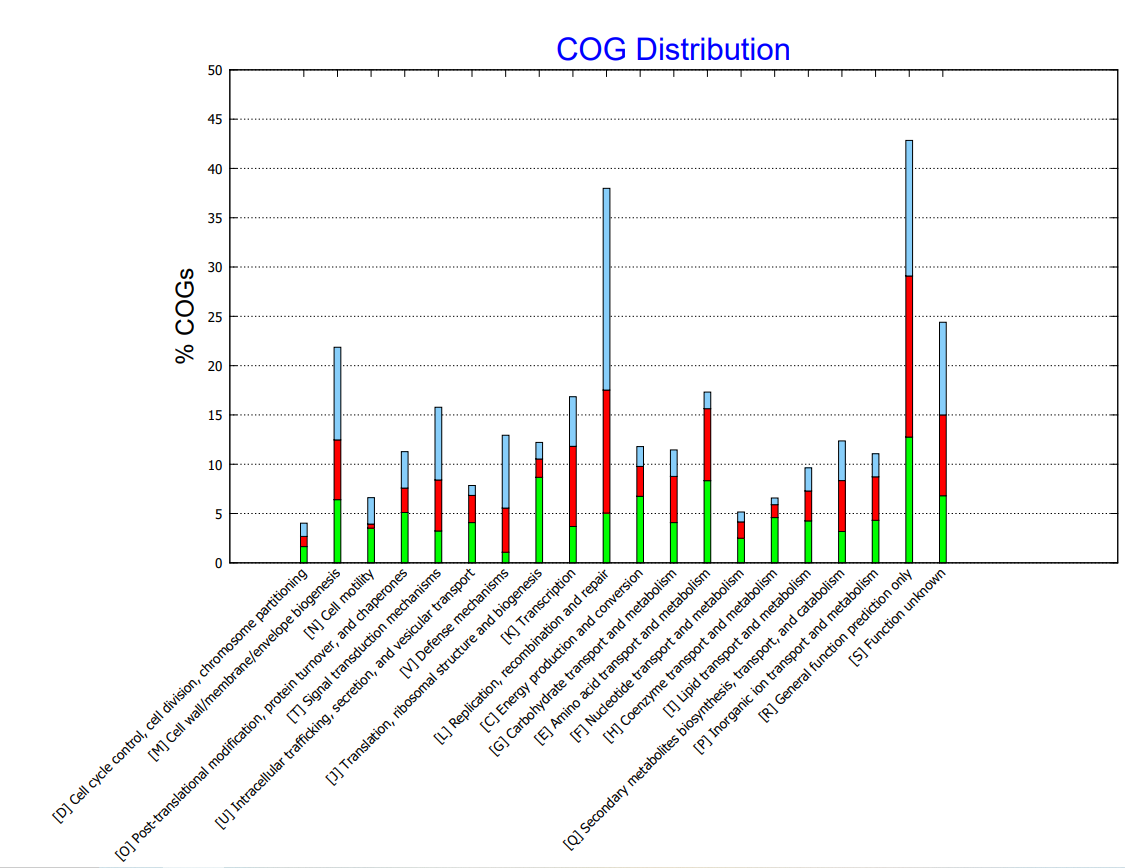


**Figure S8:** Functional distribution of Legionella pneumophila core, accessory and unique as determined by genes Clusters of Orthologous Groups (COG) analysis embedded in Bacterial Pan Genome Analysis Pipeline (BPGA). Strains included in the pangenome analysis include 26 isolates sequenced in this study most closely related to L. pneumophila and 106 L. pneumophila closed genomes downloaded from NCBI .

**Table S5:** The point mutations previously associated with antibiotic resistance in Legionella species. The mutations were aligned to E. coli K12 corresponding proteins to follow the E. coli coordinates.

| **gene** | **Antibiotic** | **Mutation on the protein level** | **Detected/non detecetd** |
| --- | --- | --- | --- |
| **gryA** | fluoroquinolone | T831I, | **ND** |
|  |  | D87N | **ND** |
| **gryB** |  | S464Y | **ND** |
|  |  | D424 | **ND** |
| **parC** |  | G77D | **ND** |
| **rpoB** | Rifampin | Q528L | **ND** |
|  |  | H541T | **ND** |
|  |  | S537P | **ND** |
|  |  | D531N | **ND** |
| **rplD** | Macrolide | G66D | **ND** |
|  |  | Q62D | **ND** |
|  |  | G64E | **ND** |
|  |  | T65K | **ND** |
| **rplV** |  | K90M | **ND** |
|  |  | G91D | **ND** |
|  |  | P87L | **ND** |

**ND-** Not detected

**
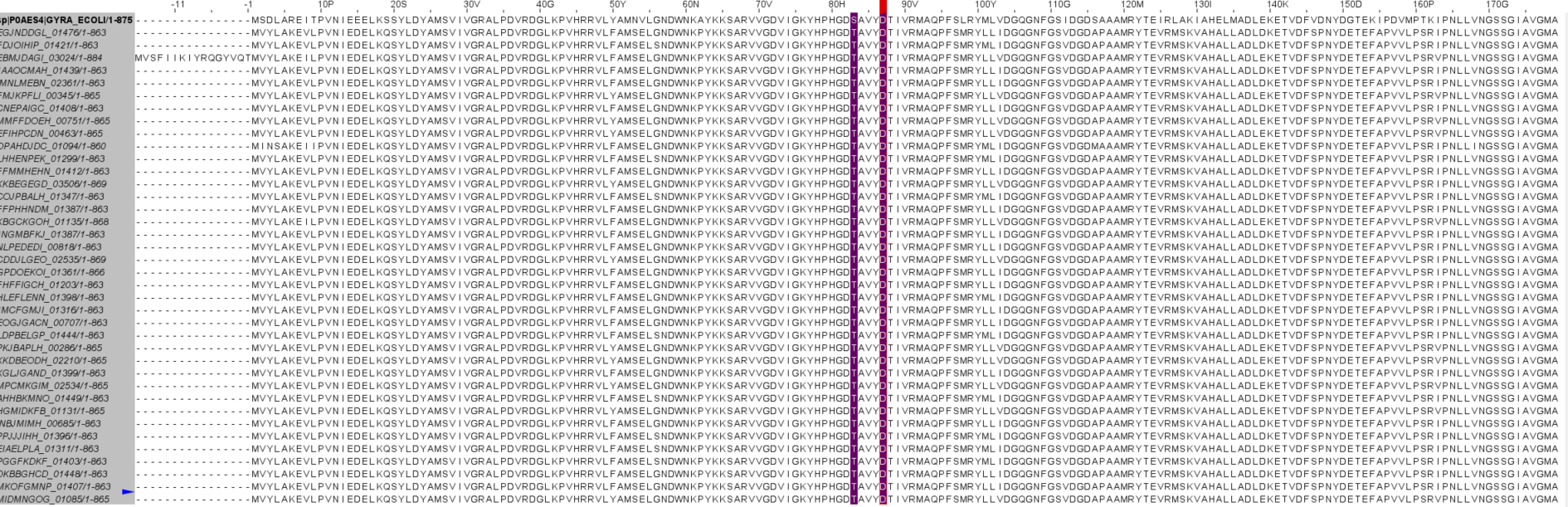
**

**Figure S9:** protein sequence encoded by gyrA gene of all isolates from this study aligned to E. coli K12. The position where the mutation was previously found is coloured in purple

**
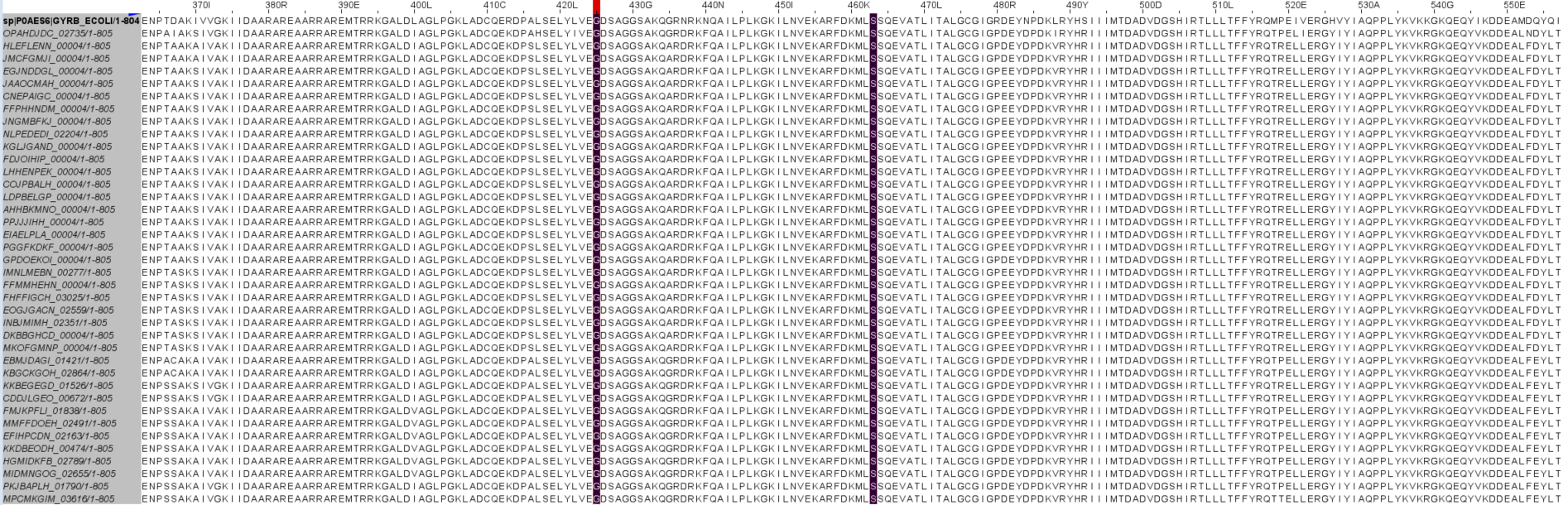
**

**Figure S10:** protein sequence encoded by gyrB gene of all isolates from this study aligned to E. coli K12. The position where the mutation was previously found is coloured in purple

**
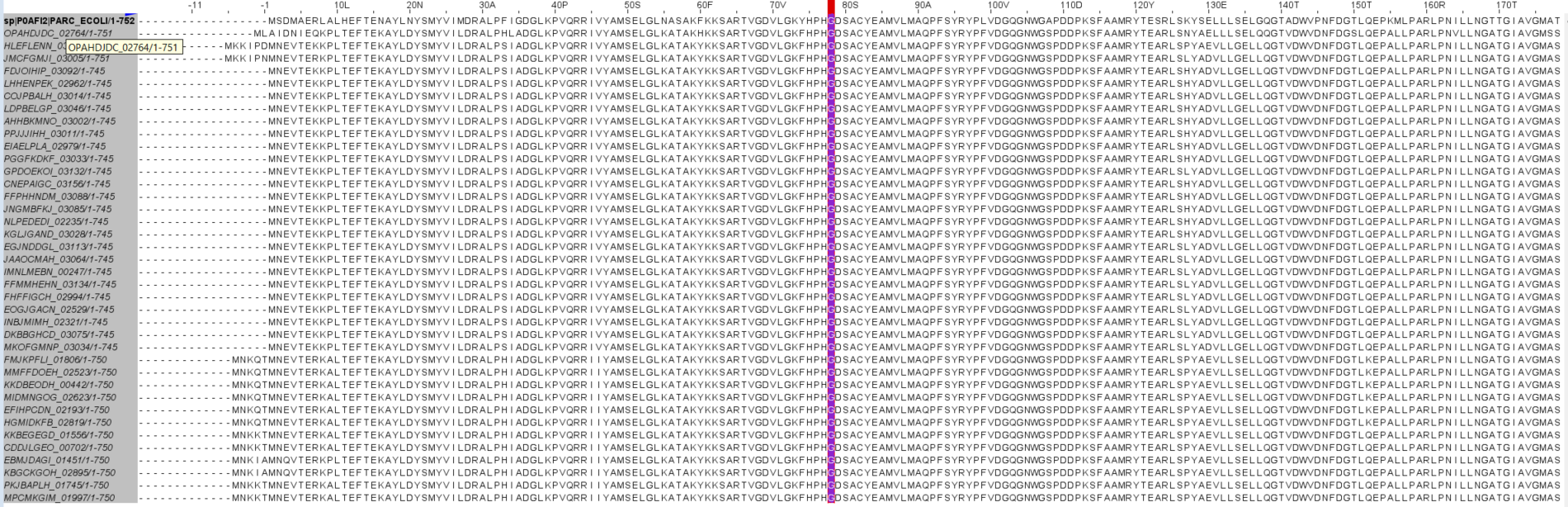
**

**Figure S11:** Protein sequence encoded by parC gene of all isolates from this study aligned to E. coli K12. The position where the mutation was previously found is coloured in purple

**
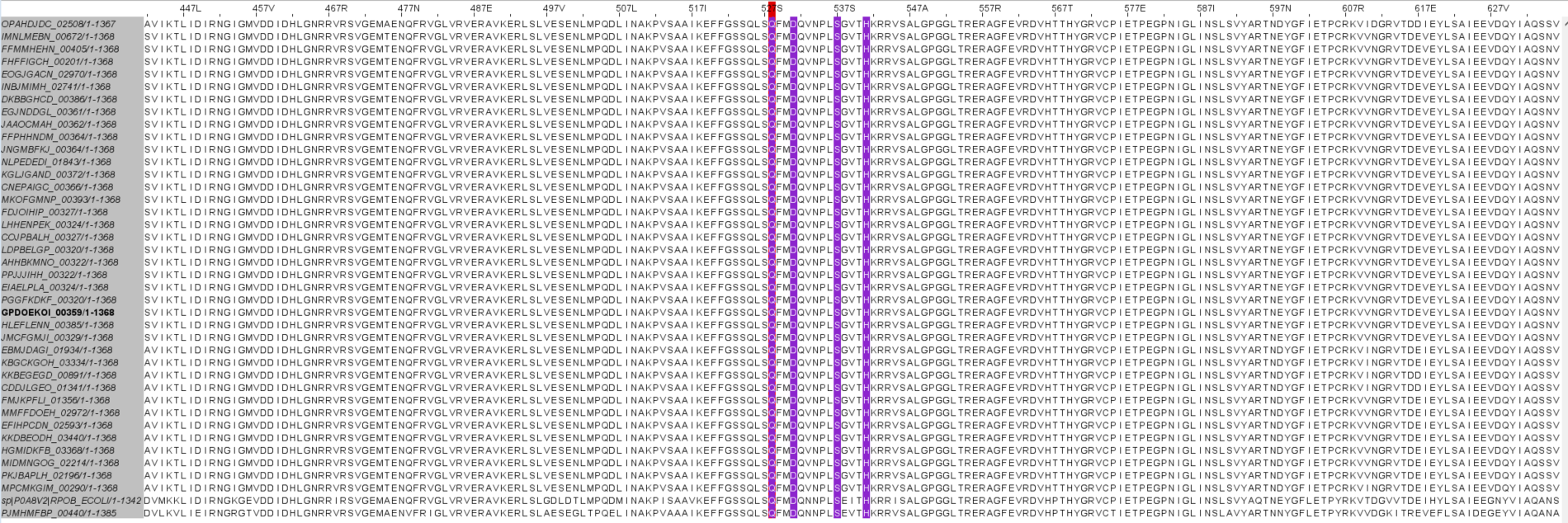
**

**Figure S12:** Protein sequence encoded by rpoB gene of all isolates from this study aligned to E. coli K12. The position where the mutation was previously found is coloured in purple


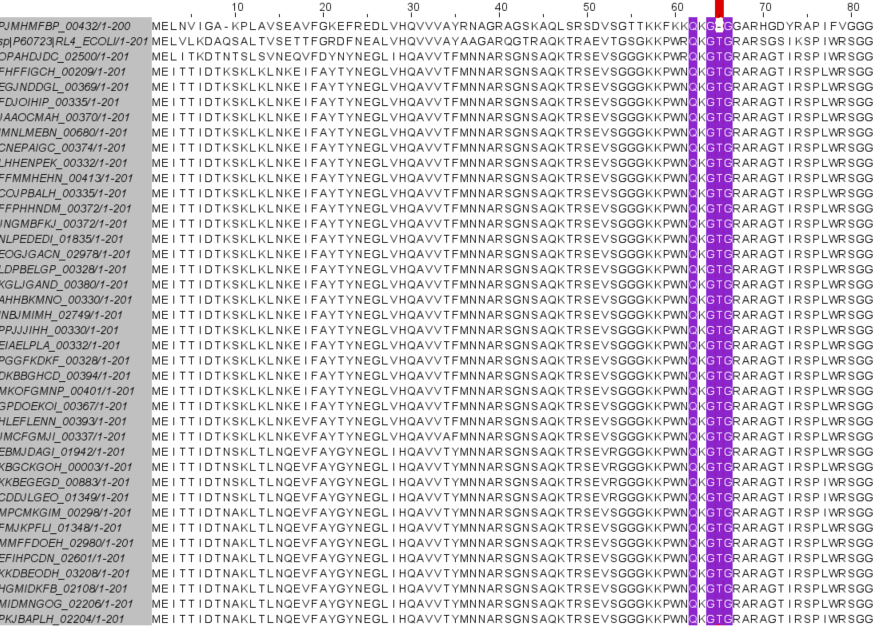


***Figure S13:*** *Protein sequence encoded by* rplD  *gene of all isolates from this study aligned to E. coli K12. The position where the mutation was previously found is coloured in purple*

**
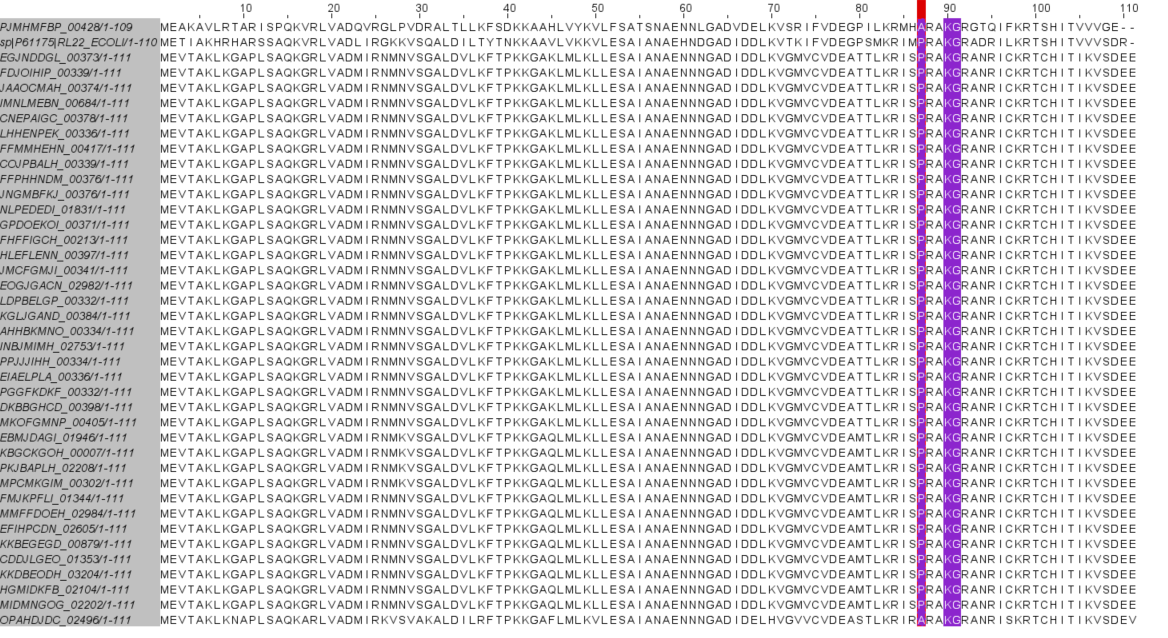
**

**Figure S14:** Protein sequence encoded by rplV gene of all isolates from this study aligned to E. coli K12. The position where the mutation was previously found is coloured in purple

**
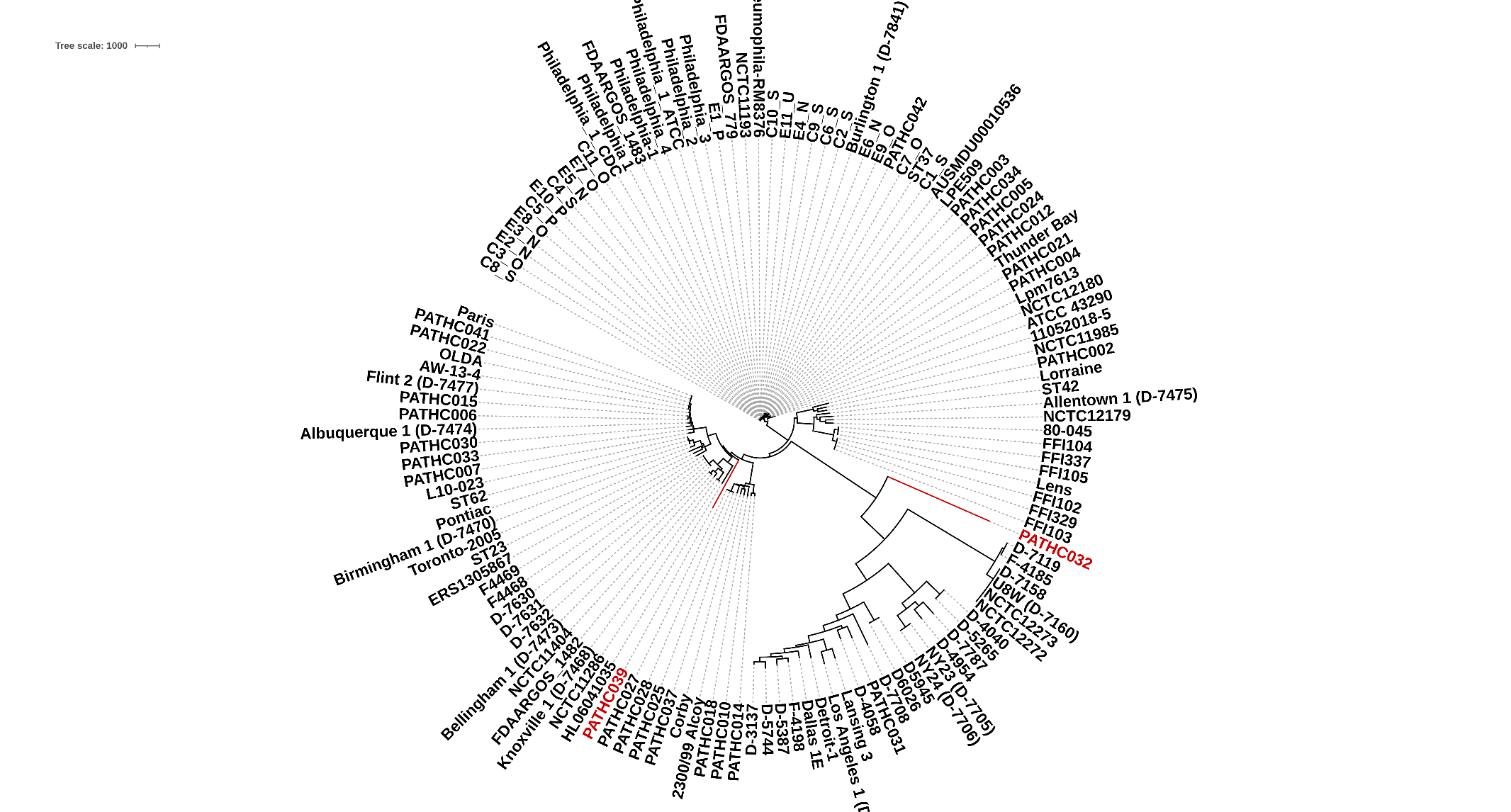
**

**Figure S15:** Phylogenetic tree based on a 25011-core-SNP matrixgenome-wide SNPs among the. A Neighbor-Joining tree was constructed using kSNP 4.0. The strains PATHC032 and PATHC039 exhibit longer branches in distinct lineages
